# Panel data evidence on the effects of the COVID-19 pandemic on livelihoods in urban Côte d’Ivoire

**DOI:** 10.1371/journal.pone.0277559

**Published:** 2023-02-01

**Authors:** Pascaline Dupas, Marcel Fafchamps, Eva Lestant

**Affiliations:** 1 Stanford University, NBER and CEPR, Stanford, CA, United States of America; 2 Stanford University, Stanford, CA, United States of America; Northeastern University, UNITED STATES

## Abstract

In early March 2020, a few cases of COVID-19 were diagnosed in Abidjan, the capital city of Côte d’Ivoire. To combat the spread of the disease, large restrictions to mobility and gatherings were introduced between mid-March and late May 2020. We collected panel survey data on over 2,500 individuals from poorer neighborhoods of the Greater Abidjan area over the period immediately before and after the start of the pandemic. We document striking drops in employment, hours worked, income, and food consumption in the first months after the onset of COVID-19, when lockdown was in place. We also find that, in response, survey respondents received more private transfers from other parts of the country, at a time when remittances from abroad fell—and that some respondents moved either temporarily or permanently. In terms of recovery, we find that subjective well-being was lower on average in December 2020 than it was at baseline. Yet, despite schools being closed between mid-March and July 2020, school enrollment suffered little: by December 2020, enrollment rates had bounced back to their baseline level. Our results finally indicate that government policies aimed at alleviating the worst effects of lockdown only reached a few people, and not necessarily those most in need.

## 1 Introduction

As the COVID-19 pandemic surged, governments around the world rushed to make decisions on how to face the health emergency. The short- and long-run impacts of the pandemic were, to a great extent, a function of the policy response to it—response taken, more often than not, with little visibility. Approaches to a pandemic that are too permissive can lead to a worsening of the health situation. Some countries were thus keen to impose mobility restrictions, expecting this would halt the spread of the virus. The potential negative effects of such measures, however, soon flooded the policy discussion. Social distancing was predicted to shrink economic activity, with poorer populations shouldering a large share of the economic consequences [1–4]. Shutting down travel and market activity were expected to hurt people economically and socially [5–7] and to result in a deterioration of non-COVID health outcomes [8, 9], including an increase in domestic violence [10]. Shutting down schools were anticipated to lead to school drop-outs and a serious loss of learning among schoolchildren [11], even in well-resourced environments [12]. As a result, there is an active concern over the policy responses to the pandemic and their longer-term effect, especially in low-and-middle-income countries (LMICs) where households are poorer and thus more vulnerable to large shocks. Documenting the channels through which the crisis affected health and economic conditions is thus key to identifying policies that remedy—rather than inflame—the situation for future pandemics.

The main contribution of this paper is to examine the multi-faceted impact of COVID-19 in a large urban and peri-urban setting. For this we chose one of the most important urban centers in West Africa: Abidjan, the largest city in Côte d’Ivoire. By the time the COVID-19 crisis hit, we had just completed a comprehensive survey of approximately 3,000 individuals from the Greater Abidjan area, conducted between December 2019 and early March 2020. To assess the impact of the pandemic, we subsequently conducted follow-up phone surveys of the same individuals in July 2020 and again in January 2021. In these surveys we asked respondents to recall key outcomes for every month over the 6 months preceding the survey. By combining this follow-up data with information from the baseline survey, we document the month-by-month evolution of employment and income, and the half-yearly changes in consumption and overall well-being over the 12 months surrounding the onset of the crisis. This is done for a large sample initially selected at random over a large number of locations in and around the city. This sets our study apart from many other efforts that had to rely on phone surveys without a well-grounded sampling frame and without detailed baseline information on the study population. In addition, we are able to follow a large number of relevant indicators, in contrast to the many studies that have information only on a subset of indicators of interest. This allows us to tell a complete story of how the population of a large African city responded to the COVID-19 pandemic and the policies put in place to stop its spread.

At the beginning of the pandemic, we find a large loss of employment and a fall in income and the number of days worked, to which corresponds a marked decrease of food expenditures. This large fall in income and consumption affects all employment categories, but the decline in food expenditures is strongest in urban slums and for respondents with precarious sources of income. We find no effect on food prices. By December 2020, the households in our sample had, on average, recovered in terms of employment, income, and expenditures. These results confirm other analyses of the pandemic in LMICs that document large decreases in income and employment during the first semester of 2020 [13–16] followed by a recovery in the second half of that year [17–19]. Regarding consumption, our urban findings confirm those found in rural settings in Kenya, Liberia, and Malawi [14, 20].

The literature has documented the different mechanisms that households used to cope with the negative economic impacts caused by the pandemic and the policy response to it. Individuals resorted to the use of savings and loans [21, 22], the reduction in the consumption of durables such as clothing and appliances [23], the reduction of gifts, remittances, and money lent to others, and the postponement of loan repayments [14]. In our study, we similarly observe that respondents reduced transfers and loans to others up to the end of our sample period, but also that they temporarily received more loans and transfers from other households in the country. In addition, a fraction of our respondents changed location, either temporarily or permanently, during this initial phase of the pandemic.

The prevalence of containment policies in LMICs has been associated with reduced mental health [9, 24–27] and hindered educational outcomes [28–30]. In line with these findings, respondents in our sample report feeling worried and having trouble sleeping more often than before the pandemic. They also report a reduced frequency of positive feelings. Children’s schooling, on the other hand, only experienced a 1% decrease in enrollment, with no significant difference between boys and girls.

Taken together, our findings document the large economic and welfare cost caused by the extension to African countries, largely untouched by COVID-19 at the time, of a worldwide attempt to ‘nip COVID in the bud’ and achieve ‘zero COVID’ globally. Given that these initial efforts ultimately failed for reasons having nothing to do with countries like Côte d’Ivoire, their net effect on West African populations was largely negative and operated via its disruption of local economies. With hindsight, the people of Abidjan would have been much better off without the lockdown policies imposed by the government, and the world would not have been worse off.

The remainder of the paper proceeds as follows. Section 2 provides background information on the course of the pandemic in Côte d’Ivoire and the government response. Section 3 describes the data used in the analysis and section 4 describes the results. Section 5 concludes.

## 2 Context: Abidjan before and during COVID-19

After a long period of turmoil, stability returned to Côte d’Ivoire after the crisis surrounding the 2010–11 election. The economy grew at an average growth rate of 5.5% per year after 2011 (Source: World Development Indicators, World Bank (2021)). In 2020, the GDP in the country was around $2325 per capita, similar to its neighbors Ghana and Nigeria. This places Côte d’Ivoire into the lower middle-income category as defined by the World Bank. Steady growth was accompanied by rapid urbanization but little reduction in the poverty rate. The tax-to-GDP ratio was at 13% in 2018, which is below the African continental average.

**Fig 1:**
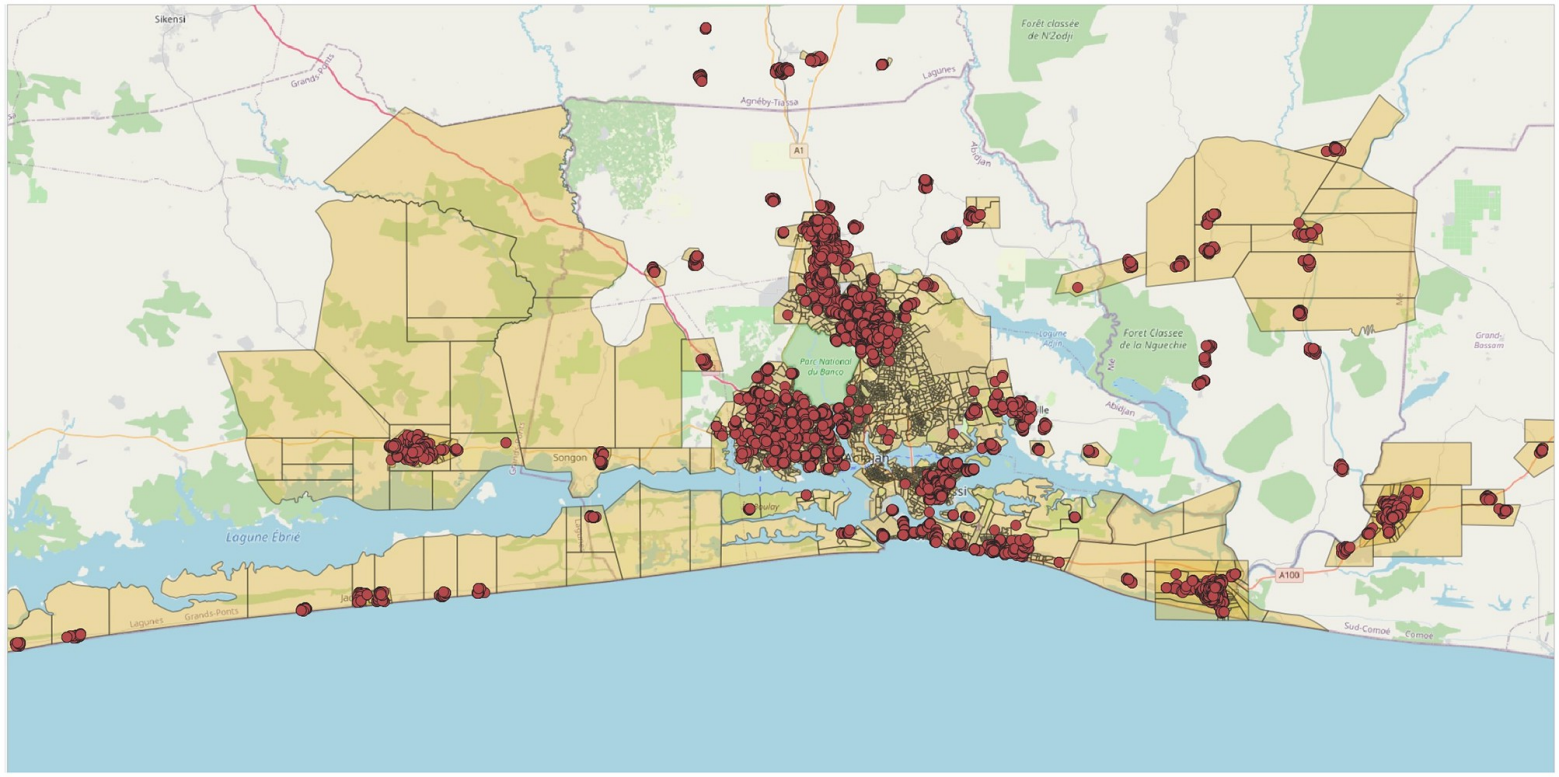
Map of the study area. The areas in pink denote municipalities included in the study. Red dots show the precise location of the surveyed enumeration areas.

The largest city, Abidjan, has a population of 6.3 million according to the 2021 census and now accounts for one fifth of the country’s population. It is an important sea port and the economic capital of the country. Its geographical coverage has been growing steadily over time, extending gradually along lagoons and islands that form the coastal area of the country in that region—see Fig 1. As a result of this particular geography, and in spite of government investment in public infrastructure, the city remains largely divided into scattered neighborhoods that are connected to the city center, but not necessarily with each other.

According to ILOSTAT, in 2017, 86.7% of the employed residents of Côte d’Ivoire were in the informal sector, which typically covers workers who are self-employed or in casual wage employment. This is one of the highest rates of informal sector employment in the world, according to the report. Recent survey data confirms that most residents work long hours and earn a low income [31]. This data also shows that while most residents have access to piped water and electricity, sewerage and garbage collection remain insufficient. The overwhelming majority of children attend school and modern medicine dominates health care provision, but access to health facilities remains constrained for those who cannot afford their fees. The government introduced a universal medical coverage card (CMU) in 2019 but, by the time COVID-19 struck in early 2020, it had only been extended to a small fraction of the population [31].

### 2.1 Health impact of COVID-19

**Fig 2:**
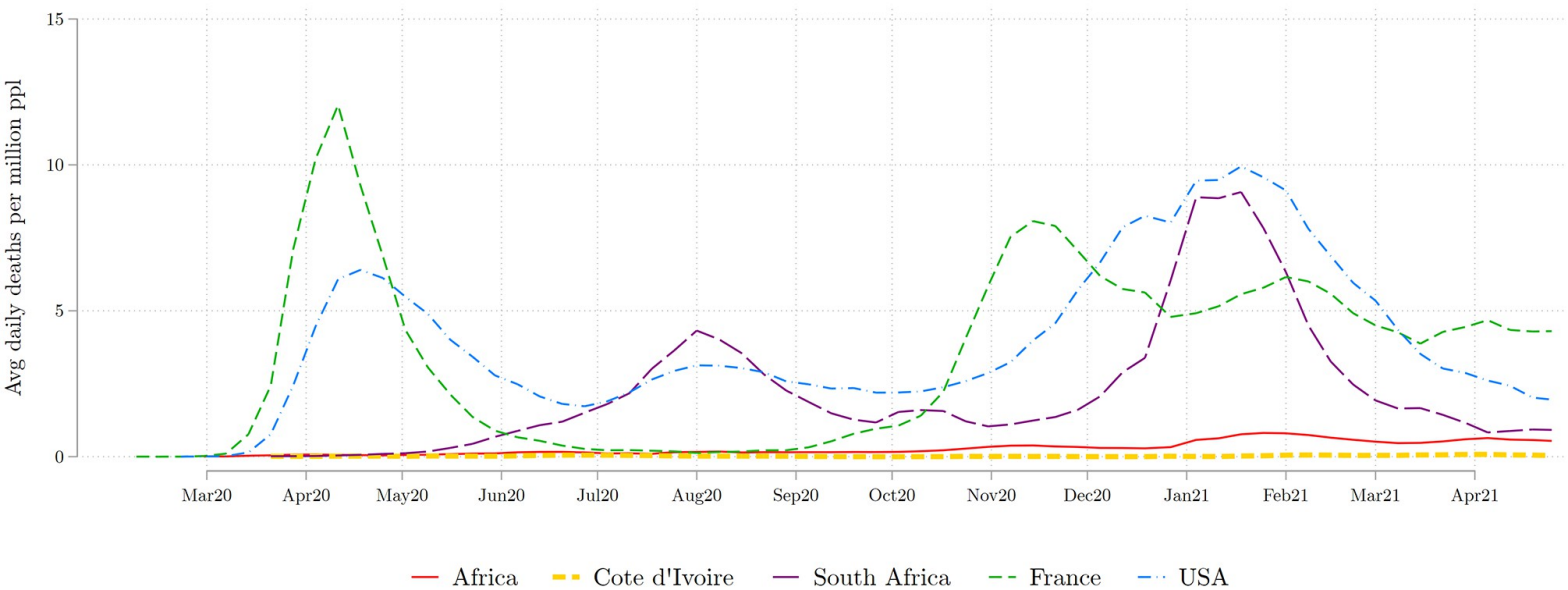
Average weekly COVID-19 deaths per million people. The Africa average does not include South Africa, which is shown separately. Source: Our World in Data.

Like much of the African continent, Côte d’Ivoire mostly eschewed the 2020 pandemic: as of the 31st of December 2020, the total number of COVID-19 deaths officially recorded in the country stood at 137 deaths for an estimated population of approximately 27 million, based on detailed world data from Our World in Data. Data on the number of cases is similarly low: by the end of 2020, Cote d’Ivoire had recorded 22,524 cases of Covid-19. In terms of evolution during the year, the first Covid wave hit Côte d’Ivoire in June 2020. Even so, the incidence of the pandemic remained very limited. Fig 2 shows the weekly average number of deaths recorded over that period for Côte d’Ivoire, compared to the United States and France, two major trade partners of Côte d’Ivoire, as well as the rest of Africa. South Africa is shown separately, given that it was much more affected than the rest of the continent. The figure shows that 2020 COVID-19 mortality in Côte d’Ivoire was several orders of magnitude lower than what was experienced in the United States and Europe.

It has sometimes been asserted that the health burden of Covid-19 in African countries has been underestimated because their health systems are supposedly ill-equipped to deal with pandemics. To verify the reliability of such claims for Côte d’Ivoire, we start by looking at the available evidence regarding Covid testing. It is true that Cote d’Ivoire only started testing for Covid-19 in mid-April 2020. But after that, the number of tests steadily increased, approaching a cumulative total of a quarter of a million by the end of 2020. It is therefore not the case that Côte d’Ivoire only tested those who had Covid.

Next, we perform simple calculations to look for other tell-tale signs of under-reporting. To do this, we rely on information from the Center for Systems Science and Engineering (CSSE) at Johns Hopkins University (JHU). According to this authoritative source, the total number of cases and deaths for Côte d’Ivoire were 86,511 and 817, respectively, as of August 24^*th*^, 2022. Using the same source, we calculate that, on August 24^*th*^, 2022, the cumulated death rate from Covid-19 had reached 0.83% of the world population while, for Côte d’Ivoire, it stood at 0.003%, which is several orders of magnitude lower. To verify whether these figures suggest that the number of cases was underestimated, we compare the ratio between the number of deaths and the number of identified cases in Côte d’Ivoire to that of the world as a whole. As a fraction of the number of identified cases, the cumulative death rate in the world on August 24^*th*^, 2022 was 1.11%, compared to 0.94% in Côte d’Ivoire. If there had been massive under-identification of new cases in Côte d’Ivoire, this ratio would be much higher. Since this is not the case, it confirms that Côte d’Ivoire was, to a large extent, spared by the pandemic.

### 2.2 Governmental response to COVID-19

#### 2.2.1 Restrictions

On March 16^*th*^ 2020, after 3 people in the country tested positive for COVID-19, President Ouattara announced restrictions including school closures, the suspension of religious services, and a ban on gatherings of more than 50 people. A week later, after another 22 people tested positive, the Ivorian government declared a state of health emergency in the capital city Abidjan. The new policy included a curfew between 9pm and 5am, a travel ban around Abidjan to prevent the spread of the virus to the rest of the country, the reduction of public transport within the city, and the closure of non-essential shops. These measures were limited to the following municipalities: Cocody, Adjamé, Treichville, Plateau, Marcory, Abobo, Attecoube, Koumassi, Port-Bouet, and Yopougon in Abidjan proper, plus the neighboring municipalities of Anyama, Bingerville, Grand-Bassam, and Songon. Apart from Cocody, Adjamé, Treichville, Plateau, and Marcory which are not included by our sample, all the other listed municipalities are within the area covered by our study. Additional government measures included financial support for hospitals. Parts of the country other than the greater Abidjan area were not subjected to any of these measures during the period of our study, and mobility restrictions appear to have been less stringent in rural areas at the edges of Abidjan.

**Fig 3:**
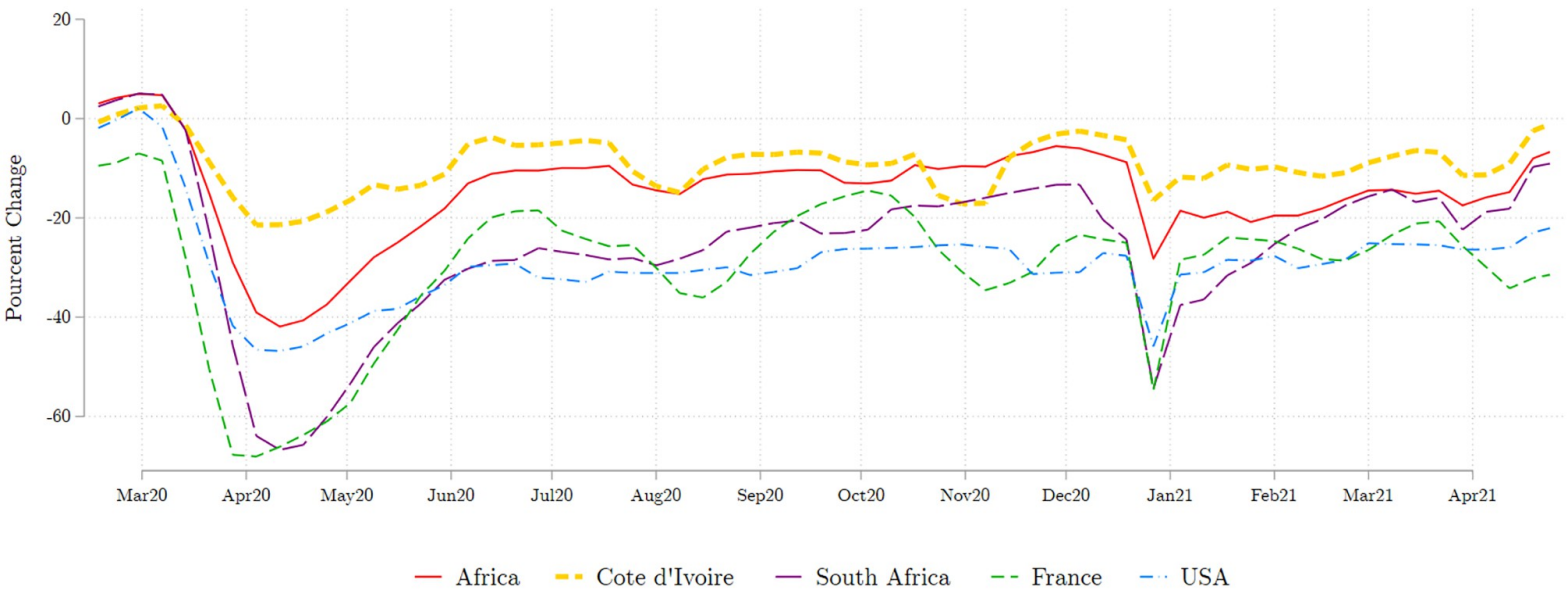
Average weekly mobility in workplaces. Baseline mobility is the median mobility for each day of the week between Jan 3–Feb 6, 2020. The Africa average does not include South Africa, which is shown separately. Source: Google COVID-19 Community Mobility Reports.

The restrictions had an immediate impact on mobility, as can be seen in Fig 3, though the reduction in mobility was muted compared to what was observed on the rest of the African continent on average. This suggests that the large effects we estimate for individuals in Abidjan may be, if anything, *underestimate* of impacts in urban areas across Africa. By the end of May 2020, most of the restrictions had been lifted, except for school closures, which remained in place until the summer vacation of 2020. As seen on Fig 3, by June 2020, mobility had resumed, but not fully, and it remained about 5% below its pre-COVID level up to at least mid-2021.

#### 2.2.2 Social programs

In April 2020, the government announced a stimulus package that included the suspension of tax payment for formal businesses, and cash transfers to the agricultural sector. To support households, the government promised to pay the electricity bills of April and May for those who were eligible for the prepaid “tarif social”. Eligible households are those with a 5 Amp service, a load barely sufficient to power a regular-size fridge. To the best of our knowledge, no other policy (such as cash transfers or unemployment insurance) was put in place to help households cope with the economic consequences of the restrictions.

## 3 Methods

As part of a long-term panel data collection initiative, we implemented an LSMS-style survey with around 3,000 individuals between December 2019 and March 2020. This baseline survey, conducted in Abidjan and surrounding areas, is part of a Stanford-funded longitudinal study involving three survey waves over six years. It is intended to provide important insights on key economic outcomes and their evolution over time in a period characterized by rapid population and economic growth. The survey was completed just before the onset of the COVID-19 crisis in Africa. IRB approval for our consent form and procedures was given by Stanford University (Protocol # 42884). The protocol was first approved on 09/29/2017, amended on 02/17/2020 and 08/31/2020, and last renewed on 08/27/2021. Verbal consent was recorded by enumerators in SurveyCTO, our survey software. It is the accepted industry standard when a large fraction of the respondents have a low level of literacy in the written language of the survey, as is often the case in less developed countries. It would be disrespectful to insist that subjects sign a paper they do not understand. This is the reason why IRB was granted for verbal instead of written consent.

### 3.1 Sampling

The area covered by this study is a large peri-urban zone surrounding the center of Abidjan which, for the purpose of this paper, we call this area Greater Abidjan. It includes all the municipalities that form the city of Abidjan proper plus the neighboring municipalities of Anyama, Bingerville, Grand-Bassam, and Songon—see Fig 1. Within this zone, we omit five municipalities that occupy the center of the city (Cocody, Adjamé, Treichville, Plateau, and Marcory) and focus on the outlying municipalities where poorer households tend to reside. This sampling frame was chosen to be broadly representative of the poorer segments of the peri-urban population of Abidjan and it contains a number of slum areas.

The sampling frame is based on a household listing that was undertaken in August 2019. This listing took place in 653 enumeration areas (EAs) randomly selected in the Abidjan City municipalities of Abobo, Attecoube, Koumassi, Port-Bouet, and Yopougon plus the municipalities of Anyama, Bingerville, Grand-Bassam, and Songon in the Abidjan district, as well as selected areas of municipalities in the periphery of the district of Abidjan (see S1 File and [31] for details). EAs are geographical units used by the National Statistical Institute for the purpose of census data collection. In densely built-up areas, each EA is calibrated to include approximately 200 households so as to facilitate the day-to-day assignment of census survey enumerators. In less dense areas, the enumerators’ workload is harder to predict because dwellings are not located in the immediate vicinity of each other. These EAs are classified as ‘rural’ by the census bureau and they contain one village typically with less than 200 households. The scattered settlement makes it possible for some households to cultivate a field or garden, but the majority work in non-farm occupations, often in the city itself. Approximately 20% of our respondents live in such ‘rural’ EAs. The rest comes from ‘urban’ EAs that are densely built-up. In the analysis that follows, we sometimes divide the sample into rural and urban respondents based on this classification. Otherwise we regard the whole sample as peri-urban because it is located at the periphery of the center of Abidjan proper.

In each of the 653 EAs, a random list of households were identified through a door-to-door listing exercise performed in August 2019 and described in S1 File. The sample for the baseline survey was constructed by first randomly selecting 70% of these listed households in each EA, and then randomly selecting one adult per selected household. To avoid oversampling individuals from singleton households, we pooled all singleton households (N = 11) together and sampled 70% of them. Because urban EAs are constructed to have roughly the same population, and in rural EAs the number of listed households is chosen to be proportional to the number of households residing there, the sample is self-weighting. This means we did not use sampling weights when randomly selecting participants among listed households.

The pre-Covid-19 baseline survey was a 4-hour, face-to-face interview. This must have created, perhaps not a bond, but at least a sense of recognition and acknowledgement of the research team as interested in the respondents’ welfare. Similar recognition need not exist in phone surveys that call people at random. The questionnaire includes a wide range of topics about the individual’s labor activities, commuting pattern, health condition, and public service access. Data collection took place between early December 2019 and early March 2020, and a total of 2,940 individuals were successfully interviewed in this baseline which, for this paper, we refer to as ‘baseline’.

During the month of July 2020, we conducted our first phone follow-up survey among the 2,691 respondents from baseline (91.5%) who provided at least one phone number in baseline. Of these 2,691 individuals, we successfully reached 2,343 individuals (87%) in wave 1.53% of those who could not be reached for the wave 1 phone survey are individuals who listed only 1 phone number in baseline. The wave 1 questionnaire includes retrospective questions about employment and income over the period from March to June 2020. It also includes short modules on consumption, prices, child schooling, and health.

A second follow-up phone survey was conducted in January 2021. In this survey, 2,406 of the selected 2,691 baseline respondents could be reached. This includes 212 individuals who were not reached in wave 1. Conversely, 150 individuals surveyed in wave 1 could not be surveyed again—leaving 85% of overlap between the two phone surveys. Overall, of the 2,691 individuals who provided a phone number in the baseline, we have *two* follow-up surveys for 2,194 individuals and at least *one* follow-up survey for 2,555 individuals (75% and 87% of the baseline sample, respectively).

The wave 2 questionnaire includes a module on employment and income similar to that used during wave 1, except that the employment module spans a longer recall period with 11 specific months between August 2019 and December 2020. The 11 months are: August 2019 when the listing exercise was undertaken; December 2019 when the baseline began; and March, April, May, June, August, September, October, November and December 2020. We also added questions on transfers that match the wording and recall periods of the questions asked in the baseline survey. We did not endeavor to push the recall period all the way to December 2018. Having such data would, in principle, have allowed us to net out naturally-occurring seasonal variation from the estimation. We did not, however, trust respondents to remember this far in the past after the traumatic events surrounding Covid and we did not want to distract them from the main focus of our study, which is to measure changes occurring during our study period of interest.

### 3.2 Characteristics of the study sample

Table 1 provides summary baseline statistics on the original baseline sample as well as on the final sample of 2555 participants who responded to at least one follow-up survey and the smaller sample of 2194 respondents who answered both waves 1 and 2 of the Covid surveys. The differences between the three samples are generally small, but we note that phone survey respondents tend to be, on average, slightly richer. This is to be expected given that we could only interview respondents with a phone, and phone ownership is correlated with income and age.

**Table 1:** Characteristics of survey respondents at baseline.

Surveyed in:	Baseline	COVID waves 1 or 2	COVID waves 1 and 2
**Demographics**			
% of females	50.5	48.5	47.2
Age	37.2	37.1	37.3
% Head of household with any secondary education	44.2	45.5	45.7
% Head of household with any tertiary education	11.3	11.7	11.5
% respondents living in their birth place	24.4	23.3	23.3
% respondent who live in rural areas	15.4	14.4	13.6
**Employment**			
% salaried or apprentices	20.9	22.3	22.5
% casual workers	12.8	13.1	13.4
% self-employed %	35.8	35.9	36.3
not employed	30.5	28.8	27.8
% All: Monthly income (x1000 FCFA)	55.8	59.4	60.2
% Income earners: Monthly income (x1000 FCFA)	73.4	76.4	76.6
**Housing**			
% renters	58.5	59.6	59.9
Monthly rent (x1000 FCFA)	27358.7	27871.8	27830.5
% respondent with gutter near the house	20.4	20.0	20.0
**Transport**			
Transport expenditure -Mondays	547.1	562.6	576.7
Transport expenditure—Wednesdays	673.7	695.4	703.2
**Personal finance**			
% owning a bank account	17.9	19.5	19.8
% owning a mobile money account	73.0	78.4	79.6
% respondents who saved in the last 12 months	43.2	44.5	45.0
**Observations**	2,940	2,555	2,194

*Notes*: The reported characteristics are those given in the baseline survey. The different columns correspond to different samples: column 1 includes all baseline respondents; column 2 includes respondents to either wave 1 or wave 2 of the COVID surveys; and column 3 includes respondents who answered to both COVID survey waves. To eliminate artifacts caused by a small number of outliers, the income data has been winsorized at the 99% percentile. Sources: baseline survey data for all reported characteristics; Covid waves 1 and 2 for sample coverage.

Focusing on the middle column, we see that 48% of respondents to the Covid surveys are female. Their average age is 37. Around 23% have never moved from their birth location and about 14% are from villages ruled by a traditional chief but located in the vicinity of Abidjan. Over a third (36%) of the Covid respondents were self-employed at baseline, 22% were either salaried or doing an apprenticeship, and 13% were casual workers. Traditional apprenticeship in Côte d’Ivoire can last for more than 5 years and people tend to continue to call themselves *apprentice* even after working for many years and taking on new responsibilities in the firm. The remainder (29%) were not collecting any income from wages or self-employment at the time of the survey.

Across the 2,555 respondents interviewed in the Covid survey waves, the average monthly income reported in baseline was around 59,000 FCFA (around $100)—roughly equal to the legal minimum wage in Côte d’Ivoire. Average monthly income among those with non-zero income was 76,000 FCFA (around 130$). Home ownership was limited to a minority: 60% of respondents were renters who on average paid 28,000 FCFA (around $48) per month in rent. Reported income refers to the individual respondent; rent is usually shared among members of the same household and most. Only 19.5% of respondents had a bank account but the use of mobile money was widespread: 78% of respondents had an account. At baseline, individuals were relying on public transportation on a regular basis, spending 3560 and 695 FCFA ($0.95 and $1.20) for transportation on Mondays and Wednesday, respectively.

To gauge whether there were differences on average between the respondents who answered the two Covid survey waves, we compared the average of baseline characteristics for those who completed the COVID survey wave 1, those who completed the COVID survey wave 2, and those who completed both. Results, not presented here for brevity, show only very minimal differences, suggesting that variation in response rates across the Covid waves is not correlated with baseline characteristics. This, however, does not eliminate the possibility that the sub-sample of respondents to the Covid surveys may be different from the 2,691 baseline individuals that we targeted, and that this difference may affect our results.

To address this concern, we ‘stress-test’ all our main findings by assuming that targeted individuals who did not respond to the Covid surveys all experienced no change from their baseline values. To illustrate how the approach works, suppose that the Covid crisis is associated with, say, a 40% fall in employment in the surveyed sample. Now imagine that 10% of the 2,691 targeted respondents failed to answer the phone surveys—and consequently we do not observe their outcomes in 2020 and 2021. What is the smallest reduction in our 40% estimate that could arise from these missing observations? The best-case scenario in when the missing 10% experienced no change and hence the smallest fall in employment that could have been measured if all targeted respondents had participated is 40% x 90% = 36%—which is still pretty large. Alternatively, the worst-case scenario is when the missing 10% all became unemployed in which case the ‘true’ mean would be 40% x 90% + 100% x 10% = 46%. This implies that the true mean must be between 36 and 46%. The reader will recognize the similarity between this approach and the Lee bounds used in standard impact analysis [32]. In the empirical section, we use this approach to evaluate the robustness of our findings to non-response bias (see S2 File).

In terms of response bias, we cannot completely rule out the possibility that people overstate the effect of Covid-19 on their lives because we are asking them about it. How serious this issue may be is difficult to assess directly, especially in phone surveys. But we do find some comfort in the fact that respondents at wave 2 report levels of employment and income in December 2020 that are not markedly different from those they report for December 2019, thus ruling out the possibility of an overall downward bias. We also note that there is broad consistency between the many effects we document, something that would be difficult to engineer on the spot for individual respondents answering a phone survey.

## 4 Empirical findings

We now use the information collected across the three survey waves to present an evolution of employment, income, and consumption over the period most affected by the restrictions introduced in March 2020. We also present information on transfers and child schooling.

### 4.1 Employment and income sources

We start by documenting the employment *trajectories* of surveyed respondents during the pandemic. This information comes from answers to questions about the main source of income for the individual respondent, not the whole household they live in. This means that the trajectories we report here concern individuals, not households. For the purpose of analysis, we categorize respondents into four mutually exclusive employment types:

- Salaried worker: an individual working regularly with the same employer at the same position and earning a regular salary paid at regular intervals. This includes workers who have a written employment contract and some who do not.
- Casual worker: an individual working episodically for different employers and either paid by day or by completed task.
- Self-employed worker: someone who derives their main income from a small business, either as a single entrepreneur or as an employer of other workers. This category includes very small businesses like selling food or water on the street, as well as a few owners of large businesses (<3% of the sample).
- Not employed: anyone not in the three previous categories. This includes individuals who report a non-labor income (individual transfers, remittances, scholarships and pensions) as their main individual income, and respondents who do not report any individual income (16%).

**Fig 4:**
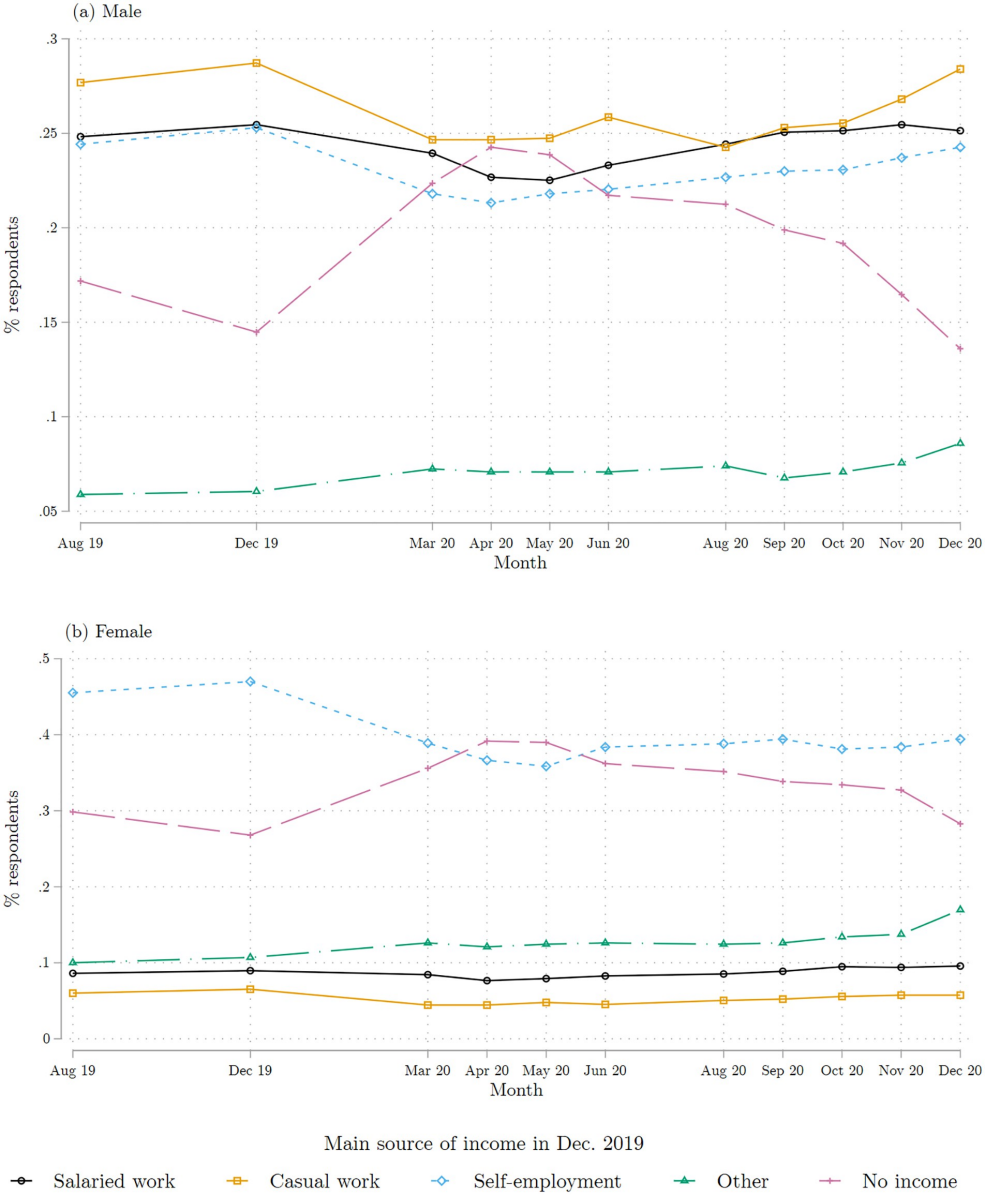
Main source of individual income, by month. (a) Males (b) Female. Main reported source of individual income for each reported month, broken down by the gender of the respondent. Salaried work includes a small number of apprentices; self-employment includes farming; and the ‘other’ category combines all unearned source incomes (e.g., transfers and income from property). The sample is limited to the respondents interviewed during the COVID survey wave 2. Source: COVID survey wave 2.

In Fig 4, we report the employment trajectory of respondents, broken down by gender and by their employment category reported for December 2019. For each gender and baseline employment category, we plot the share of people in each of the four employment categories in the months following the beginning of the pandemic. We also show where they were in August 2019, so as to benchmark what the regular turnover rate is.

While the pandemic may not have created a health crisis in Abidjan in 2020, the lockdown introduced in mid-March 2020 greatly increased the share of people who report not being employed (see Fig 4). During the full month of lockdown (April 2020), about 40% of female respondents report not being employed, compared to 28% at baseline; close to a quarter of men do the same, compared to 15% at baseline. The non-employment rate for men remained higher than pre-COVID throughout 2020, only reverting to its pre-level by December 2020. For women, non-employment remained 5 percentage points higher than pre-COVID by the end of 2020. S2 File shows that our estimates, while large, are conservative—if we assume that those who could not be surveyed all stayed employed, we find a substantial increase in non-employment similar to our estimates, but if we assume that those who attrited all lost their source of income, the shock is considerably greater.

The top panel of Fig 4 shows that approximately 12% (-3pp from a 25% base) of men employed as salaried worker at baseline lost their salaried employment. The same happened for salaried women, men and women in casual work, and for men and women in self-employment.

These changes in employment status proved remarkably persistent: over the 12 months interval covered by our wave 1 and 2 surveys, we only see a very gradual recovery for those who lost their pre-COVID employment situation. Also, during and after lockdown, we only see limited turnover between employment categories, except for exit from non-employment. By the end of our panel survey in December 2020, 25% of women and 20% of men who were not employed at baseline had found a source of labor income, mainly in casual work for women and self-employment for men.

**Fig 5:**
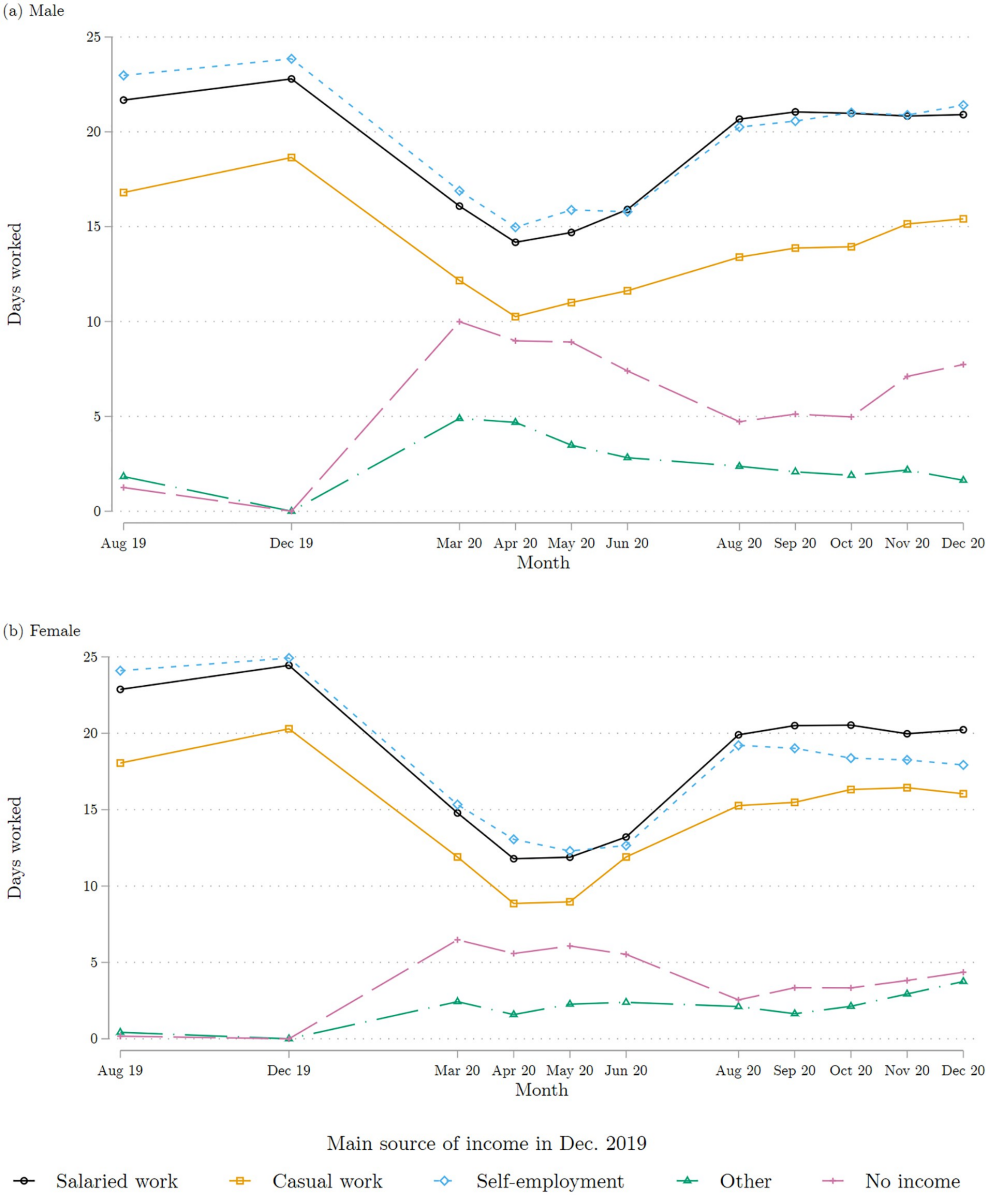
Days worked each month, by main income source in December 2019. (a) Males (b) Female. Each marker represents the average of the reported number of days worked in each reported month, broken down by the main source of individual income reported for December 2019. The top panel combines all the male respondents interviewed in either of the two COVID survey waves 1 and 2; the bottom panel does the same for female respondents. Source: COVID survey waves 1 and 2.

Fig 5 depicts the number of days worked by individuals per month, broken down by income source at Baseline. Mirroring the employment trajectories mentioned above, there is a large drop in the number of days worked per month during the first semester of 2020 for those individuals who were salaried workers, casual workers, and self-employed in December 2019.

Geographically, the maps in S1 Fig show that all sampled areas were affected. The proportion of individuals reporting labor income dropped everywhere up until July 2020. Recovery seems uneven, however: by December 2020, some areas (e.g., Yopougon, Bingerville) had recovered their pre-covid levels of employment, but this is not the case in others (e.g., the south of Abobo).

### 4.2 Income

**Fig 6:**
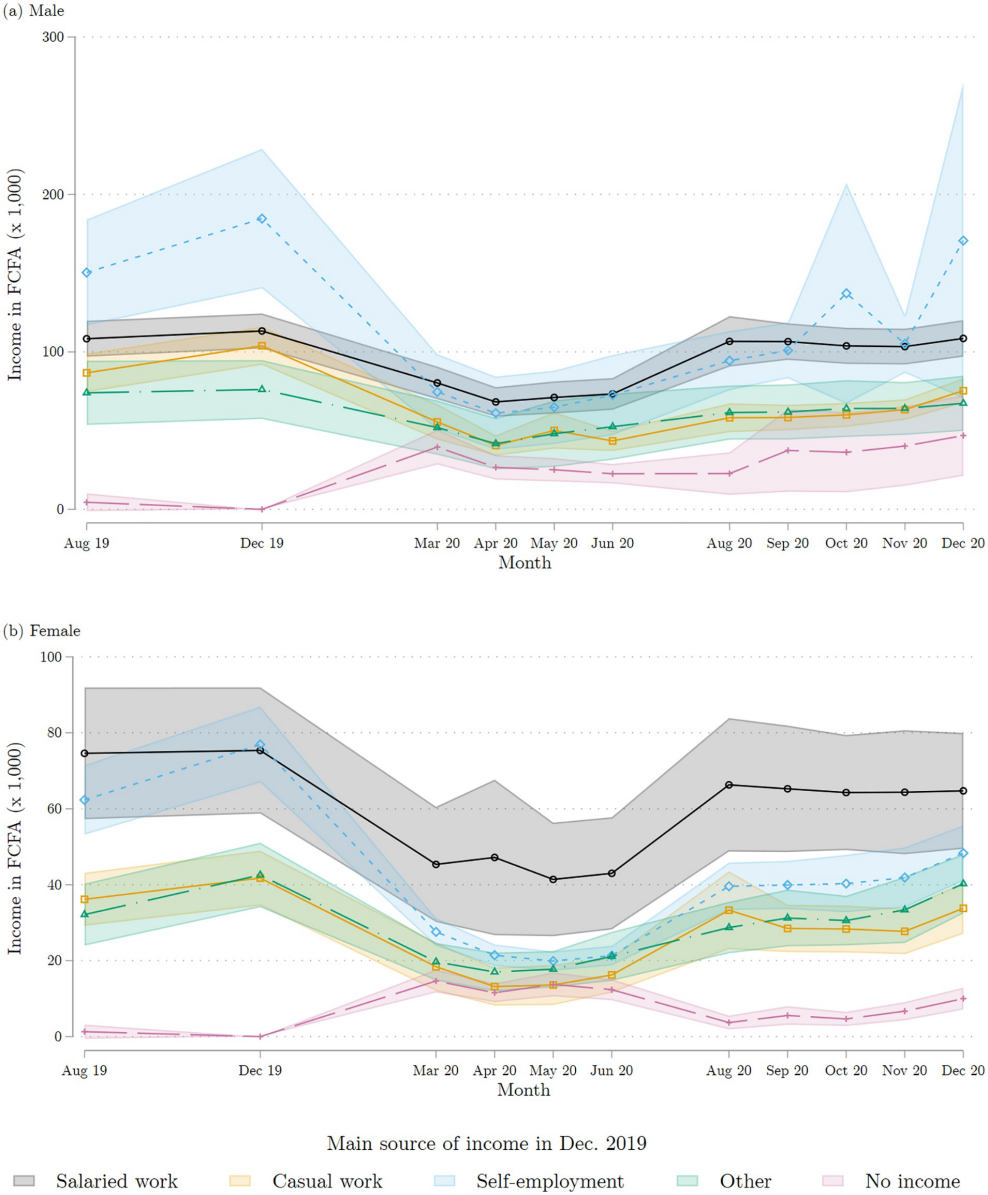
Individual income each month, by main source of income in December 2019. (a) Males (b) Female. Each marker represents the average of the reported individual income in each reported month, broken down by the main source of individual income reported for December 2019. Each average is calculated by regressing the reported monthly individual income on monthly dummies. The corresponding 95% confidence intervals are shown in color. The top panel combines all the male respondents interviewed in either of the two COVID survey waves 1 and 2; the bottom panel does the same for female respondents. Source: COVID survey waves 1 and 2.

Fig 6 shows the evolution of income in the first year of the pandemic. Here again, we group individuals based on their baseline employment situation. In line with the employment trajectories described above, we observe large drops in earned income for the four months of March, April, May and June 2020, followed by a recovery in August 2020. Note that for men, we observe a kink in October 2020, which correspond to the Presidential election, a period full of uncertainty during which many shops closed. For those who were in salaried employment in December 2019, average income levels at the end of 2020 are broadly similar to their pre-COVID figures. Individuals who were casual wage workers and self-employed individuals at baseline see, on average, a marked but incomplete recovery in their income levels. These patterns are similar for both men and women, but at quite different levels of average income. Furthermore, women seemed to have recovered relatively less by December 2020. We also see that individuals who were not reporting any income in December 2019 experienced an income increase on average, albeit to a low level. Overall, there was recovery but the recovery was slow, especially for males who were initially employed as casual workers.

**Fig 7:**
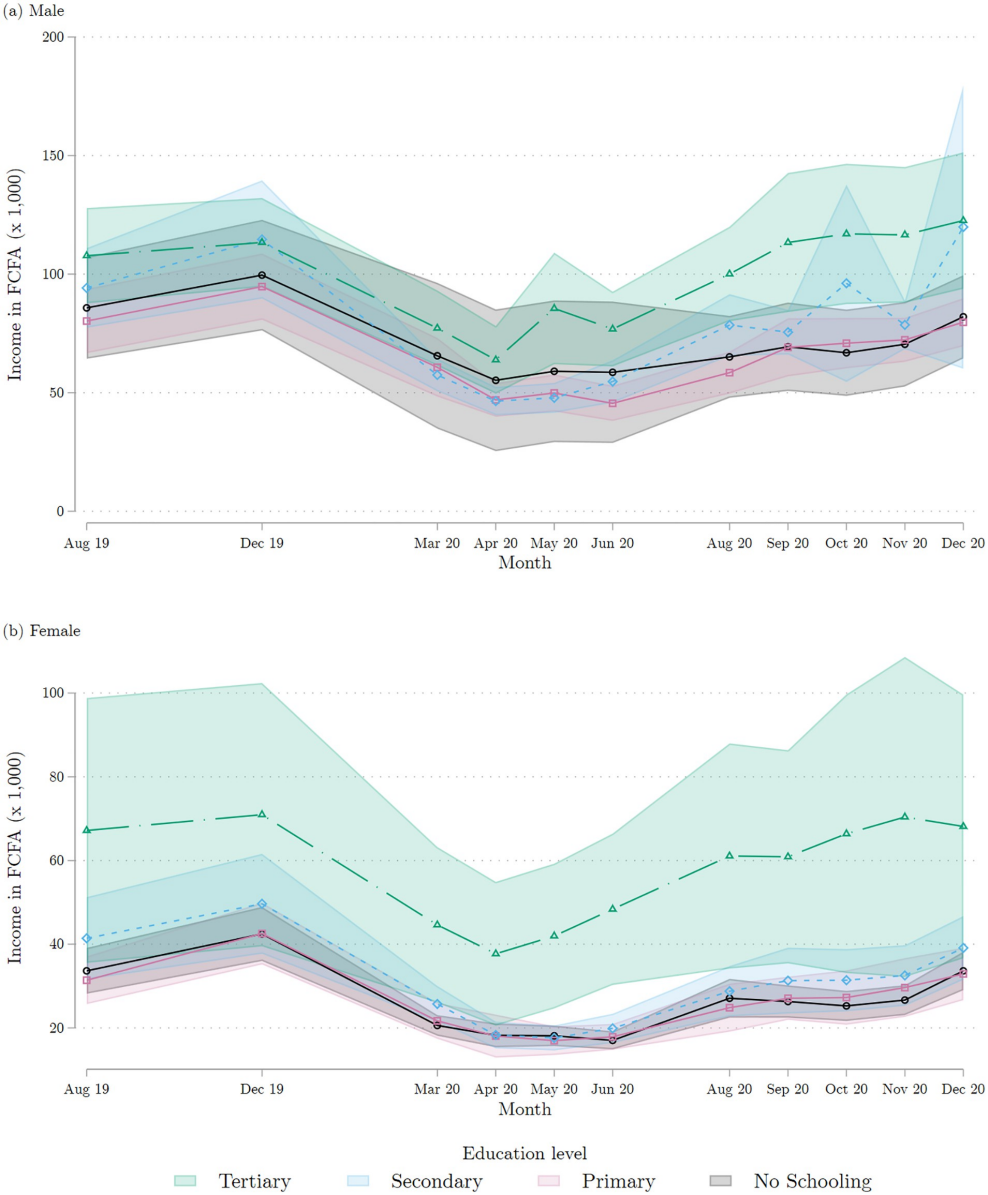
Individual income each month, by educational attainment at baseline. (a) Males (b) Female. Each marker represents the average of the reported individual income in each reported month, broken down by the respondent’s educational attainment at baseline. Each average is calculated by regressing the reported monthly individual income on monthly dummies. The corresponding 95% confidence intervals are shown in color. The top panel combines all the male respondents interviewed in either of the two COVID survey waves 1 and 2; the bottom panel does the same for female respondents. Source: COVID survey waves 1 and 2.

Fig 7 decomposes the income trajectory during the pandemic by education level. All groups, irrespective of education, were severely impacted. Even for respondents who completed tertiary education, we observe during the March-June period a 40% and 50% decline in income for men and women, respectively. This drop is similar in magnitude to that incurred by respondents with secondary and lower level of education (44% and 50% for men and women, respectively). High education therefore does not seem to have protected workers against the income shortfall—a finding that is especially true for women.

**Fig 8:**
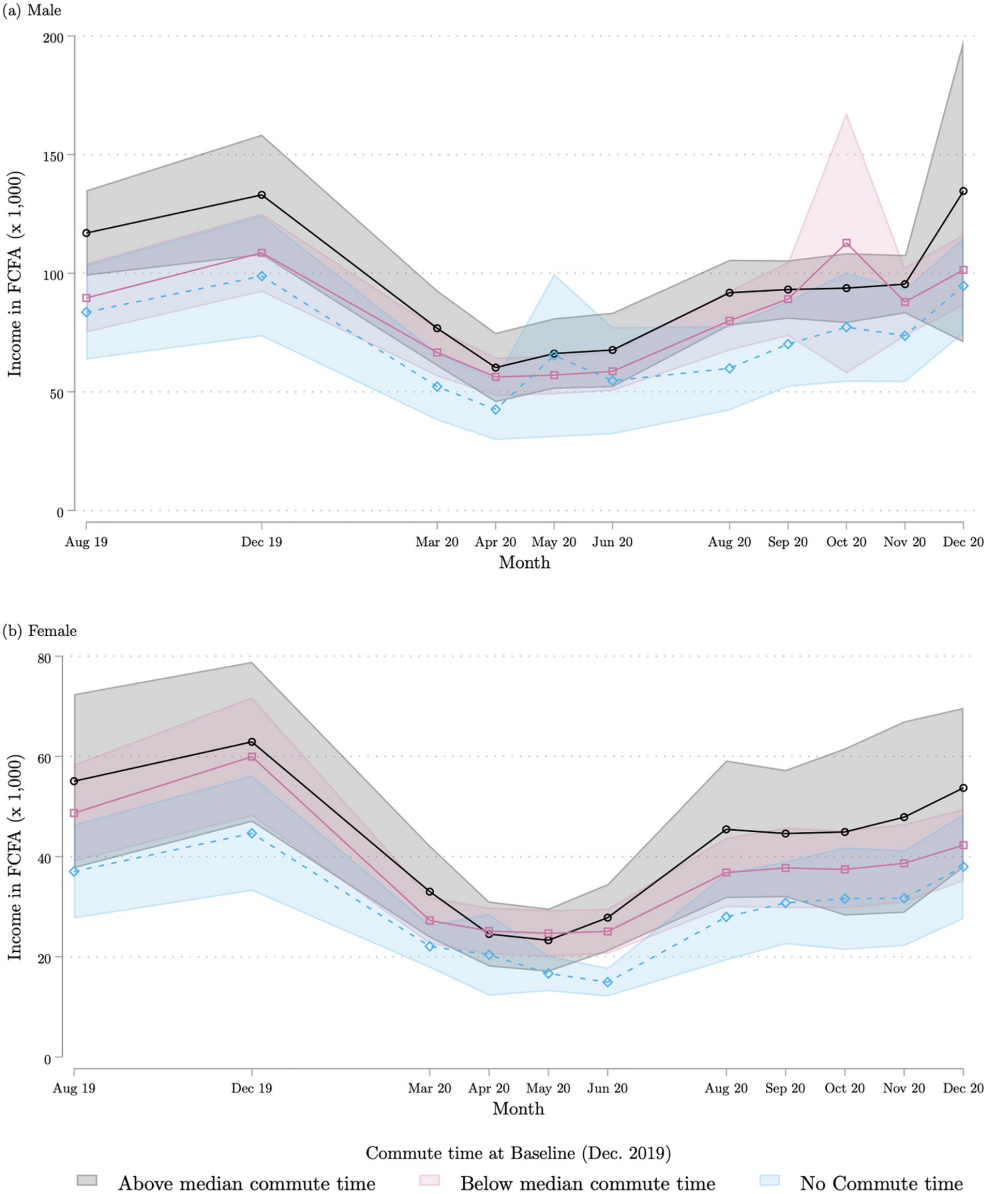
Individual income each month, by commute time at baseline. (a) Males (b) Female. Each marker represents the average of the reported individual income in each reported month, broken down by the respondent’s commute time at baseline. Commute time is divided into three categories: zero commute time (this includes non-working individuals and individuals working from home); below median non-zero commute time; and above median non-zero commute time. Each average is calculated by regressing the reported monthly individual income on monthly dummies. The corresponding 95% confidence intervals are shown in color. The top panel combines all the male respondents interviewed in either of the two COVID survey waves 1 and 2; the bottom panel does the same for female respondents. Source: COVID survey waves 1 and 2.

The second dimension of heterogeneity we examine is baseline commute time. This serves to test the hypothesis that respondents who had a longer commute at baseline were more affected by travel restrictions. Fig 8 divide the sample between respondents who have a commute time above the median (20 minutes); respondents with a below-the-median but non-zero commute time; and respondents with no commute at all. Results show that all groups experienced a large drop in income between March 2020 and June 2020. The lack of difference may be driven by the fact that commute time is also highly correlated with having a salaried job located in a central part of the city, away from the low socioeconomic-status neighborhoods of our sample, and that salaried workers suffered a large income drop during the early stages of the pandemic. Put differently, restrictions to mobility tended to penalize individuals with longer commute times; but they also penalized salaried workers more on average. Because salaried workers tend to live closer to the city center, these two factors happen to cancel each other out in our data.

### 4.3 Consumption expenditures

Since it was not possible to administer a detailed consumption module over the phone, we rely on expenditures as a proxy for consumption.

**Fig 9:**
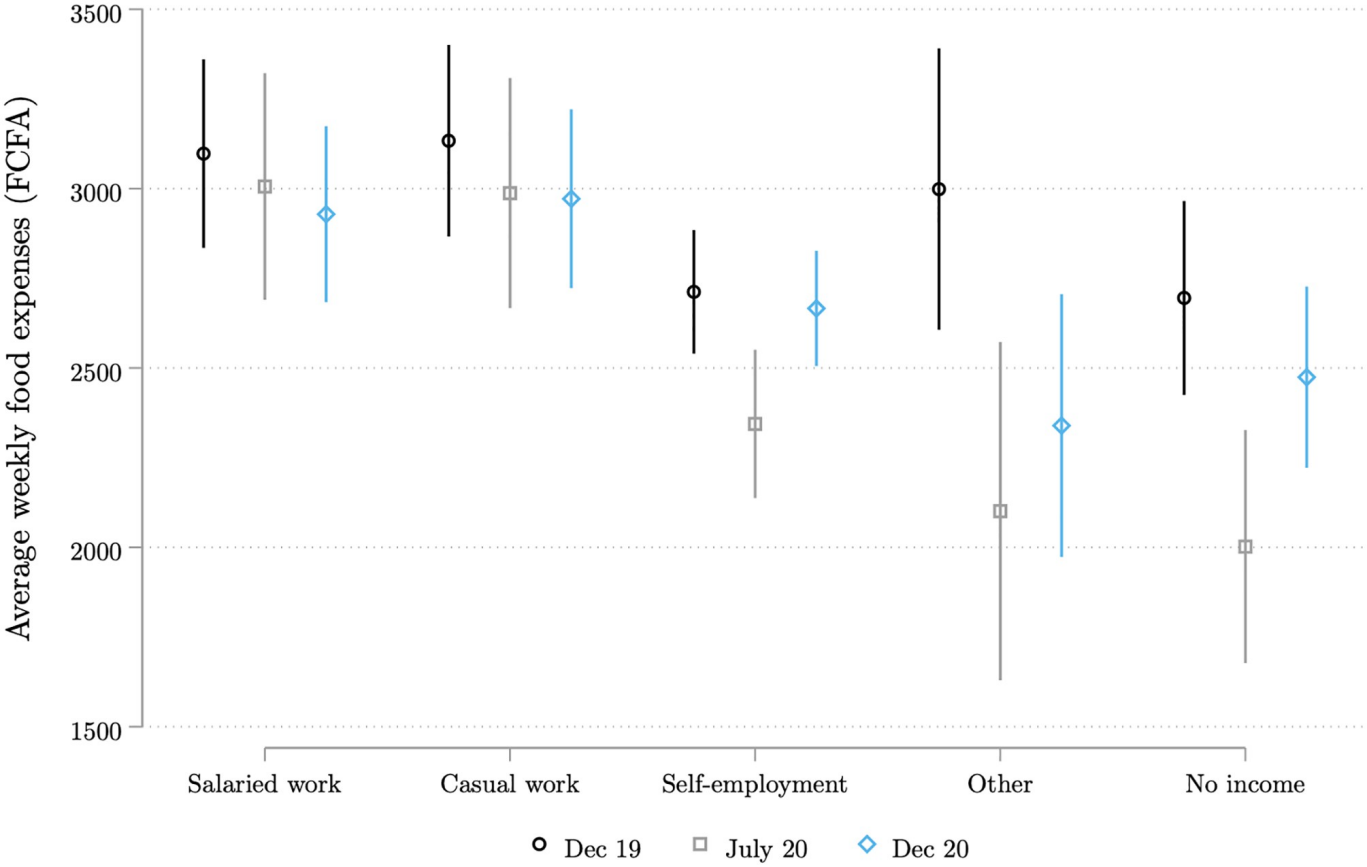
Household weekly expenditures per capita on food, by main income source in December 2019. Each whisker plot presents the average of the household weekly expenditures per capita reported in each of the three surveys, broken down by the respondent’s main source of individual income for December 2019. Each average is calculated by regressing the reported expenditures on survey dummies. The corresponding 95% confidence intervals are shown in color. To correct for outliers, the top 1% of the weekly expenditure was winsorized. The Figure combines all the respondents interviewed in either of the two COVID survey waves 1 and 2. Source: Baseline survey and COVID survey waves 1 and 2.

**Fig 10:**
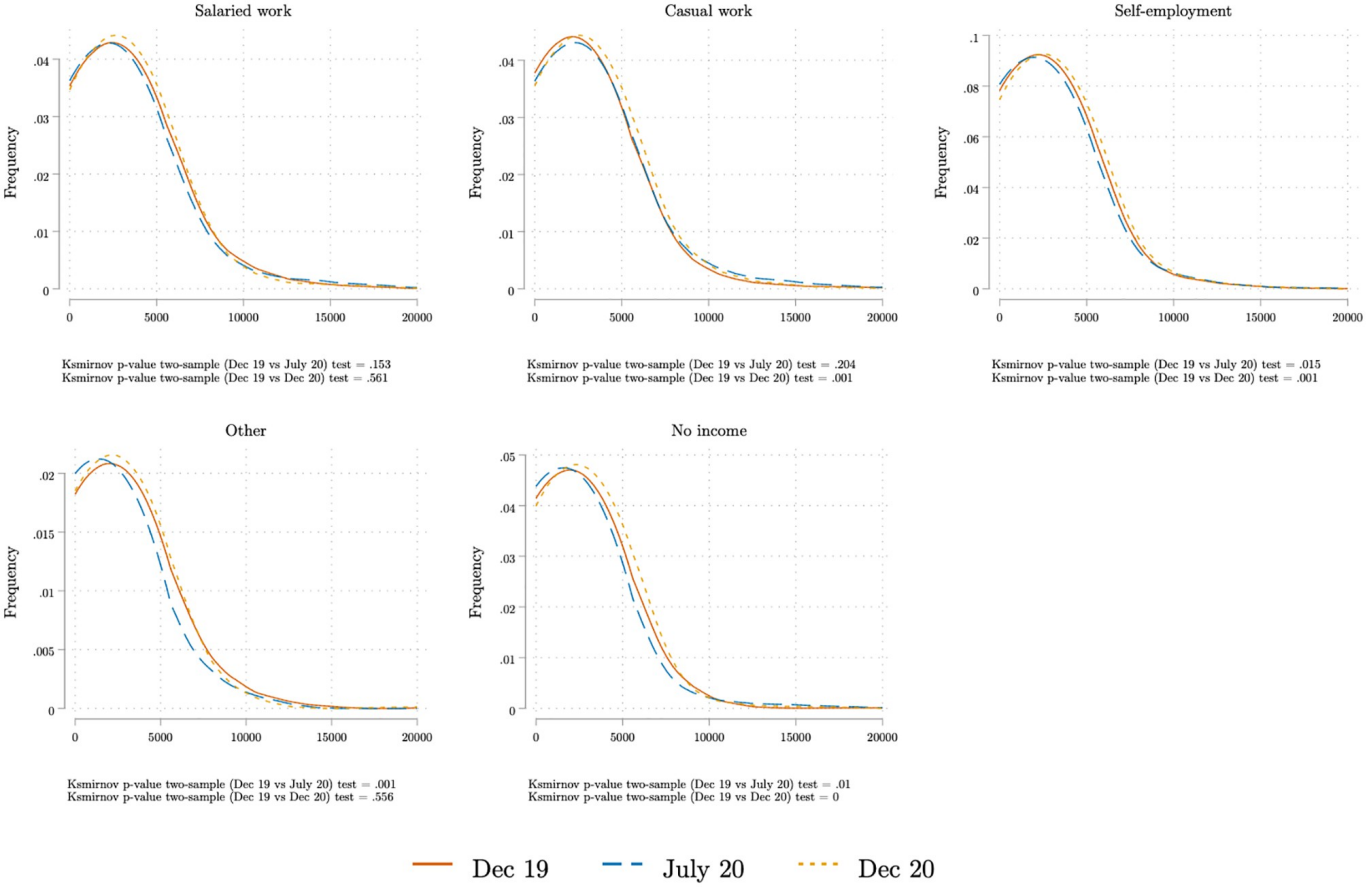
Kernel density of food expenditures per capita, by main income source in December 2019. The Figure shows the estimated Kernel density of the household weekly food expenditures per capita reported in each of the three surveys. Each panel only includes respondents reporting a particular main source of income for December 2019. To correct for outliers, the top 1% of the weekly expenditure was winsorized. The Figure combines all the respondents interviewed in either of the two COVID survey waves 1 and 2. Source: Baseline survey and COVID survey waves 1 and 2.

We asked respondents, regardless of their relationship with the head of household, how much money the household spent on food in the seven days preceding the survey. Fig 9 shows the implied average consumption expenditure per capita in each survey round (baseline, COVID wave 1 and wave 2). We use the total number adults and children in the household as the denominator when calculating per capita expenditure. We see a clear drop in consumption in July 2020. By December 2020, however, expenditure levels had returned to their pre-COVID level, except fot the ‘other’ category which includes non-earned income. To see whether the fall in expenditure is larger for high or low expenditure households, Fig 10 plots the kernel density of expenditure per capita for each survey wave. We compute Kolmogorov-Smirnov tests of the equality of the distributions across time periods. The probability density functions for December 2019 and December 2020 are nearly identical. But for July 2020 (the dashed line), we observe a clear shift to the left—i.e., a fall in expenditures per capita—for all employment categories.

Respondents to wave 1 and 2 surveys also answered qualitative question about food consumption. In the wave 1 survey a non-negligible proportion of respondents reported difficulties: 35.2% stated being unable to afford their habitual food consumption because of high prices; 43.3% report reducing food expenditures due to lack of cash; and 36.2% report reducing the number or size of their meals. By wave 2, these numbers had fallen significantly to 25.2%, 33.9% and 22.2%, respectively. These responses confirm the large negative effect that lockdown had on food consumption.

Next, we look at the evolution of consumption expenditures across space—see S2 and S3 Figs. We find that food consumption expenditures per capital fell in all three spatial categories, but urban dwellers—who at baseline enjoyed a higher level of food consumption—were hit the hardest. This is particularly true of urban slum dwellers, who experienced a decrease in food consumption of more than 20% in mid-2020.

**Fig 11:**
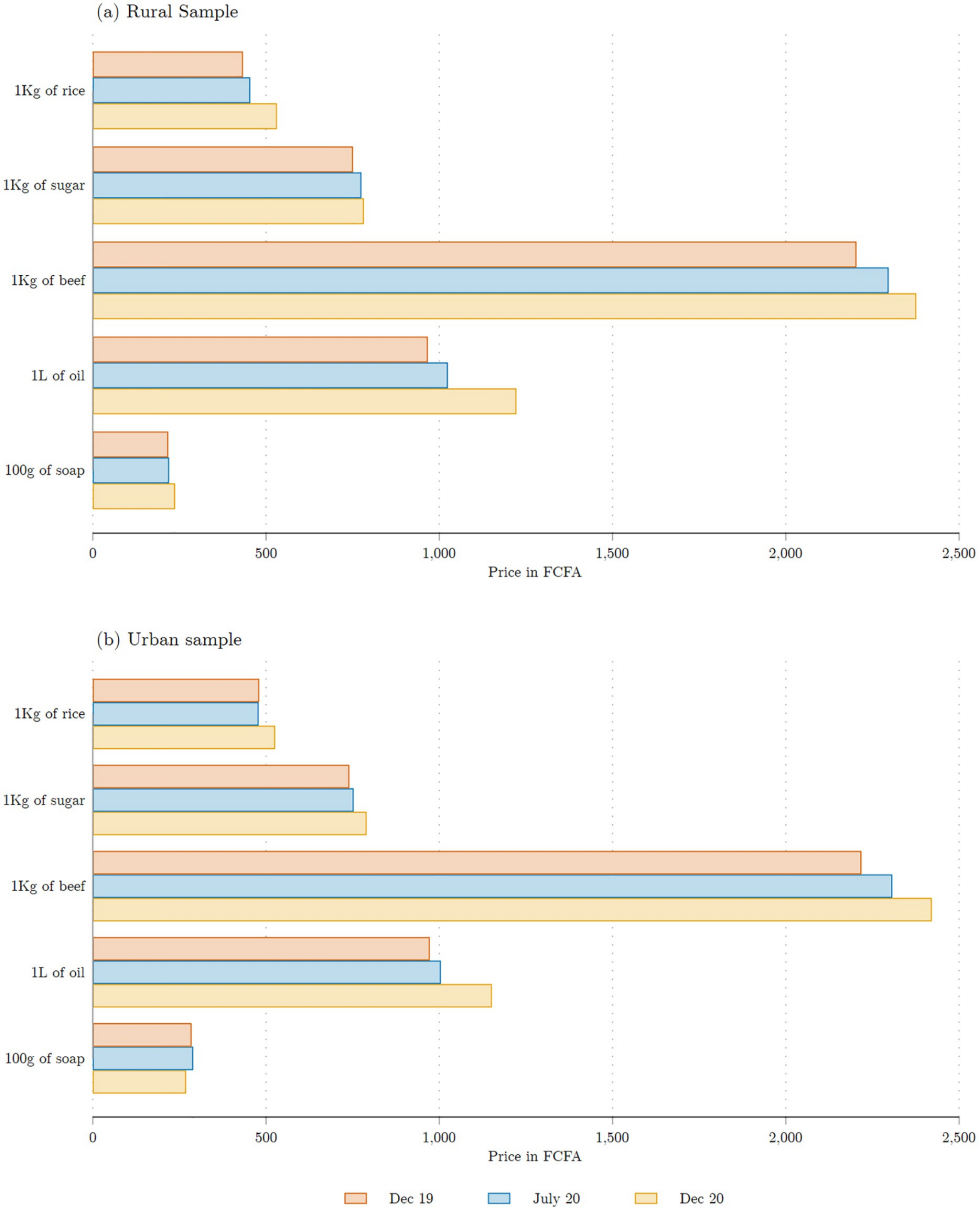
Food prices. Each bar represents the average price reported by respondents for each purchase category in each of the three surveys: baseline (for December 2019), COVID wave 1 (for July 2020) and COVID wave 2 (for December 2020). The sample includes 1,235 respondents randomly selected from all sampling areas to answer these questions. Source: Baseline survey and COVID survey waves 1 and 2.

Could the large reported changes in food expenditures reflect variation in prices? To investigate this, we report information about changes in prices as reported by respondents, broken down between the urban and (peri-urban) rural areas. Respondents were asked to report the price of five commonly purchased items in December 2019, July 2020, and December 2020. The five items are: 1Kg of rice, 1Kg of sugar, 1Kg of beef meat, 1Liter of oil; and 100g of soap. Fig 11 indicates that the first year of the pandemic was associated with only a limited increase in prices, and that reported price changes were virtually identical in our urban and rural study areas. Across the entire sample, the average reported price increase between December 2019 and December 2020 is 1.8%, a figure comparable to the 2.4% national inflation rate estimate reported by the World Bank for 2020.

### 4.4 Mobility and relocation

In wave 1, we asked respondents whether they increased or reduced their movements during lockdown. Only 1.2% report increasing their movements; 22.4% report no changes and 76.4% reduced their movements. 41.5% of wave 1 respondents also report an increase in the cost of transport during lockdown. These answers demonstrate the effect that the pandemic had on the mobility of the study population. Of those who reported reduced mobility, 90.8% list fear of the virus as a reason while 38.1% mention lack of work and 3.3% the cost of transport.

We asked all wave 1 and 2 respondents whether they moved since the previous survey. By July 2020, a sizeable proportion of respondents (8.3%) report having relocated from their baseline location. Of those, 2% reported the move as permanent; the rest (6.3%) reported it as temporarily. We also find that 6.9% of respondents listed moving between COVID survey waves 1 and 2—5.1% report the move as permanent and 1.8% as temporary. In total across the two COVID waves, 10.6% of respondents report relocating once—temporarily or otherwise—and 2.0% relocated twice. Across the two waves 5.7% of respondents report moving permanently once and 0.6% twice. While we do not have strictly comparable information on short-term movements before the pandemic, these numbers look high relative to relocation frequency before COVID [31].

We also collected information on the reason for the move. Most wave 1 and 2 respondents give answers similar to those given for moving across locations before COVID, namely following one’s family (28% compared to 48% at baseline), starting a new occupation (25% compared to 13% at baseline), or looking for work (8.6% compared to 10.2% at baseline). Some answers do stand out, however. Fewer respondents to the two COVID surveys report moving in order to study or get married (2.5% each compared to 10.8% and 9.1% at baseline, respectively). But more COVID survey respondents (9.2% compared to 1.0% at baseline) report moving to provide family help (14.1% in wave 1), suggesting they are assisting another household during the crisis; and 4.1% moved because they could not pay rent. Some responses are specific to the wave 1 survey: 5.9% say they moved to flee the coronavirus and 7.7% because they cannot reenter Abidjan.

Taken together, these findings suggest that the restrictions imposed in the early months of the pandemic caused significant disruptions to respondents’ choice of residence. We also note that respondents who relocate temporarily report 25% lower consumption than non-movers, while those moving permanently report 12.5% lower consumption than non-movers on average. While these correlations are not causal, they nonetheless indicate that temporary relocation in the wake of the pandemic is associated with facing hard times.

### 4.5 Monetary transfers and government support

Next, we examine how respondents coped with the changes in employment and income that occurred during the first year of the pandemic. We provide evidence on two of the most relevant coping mechanisms: private transfers and government transfers.

**Fig 12:**
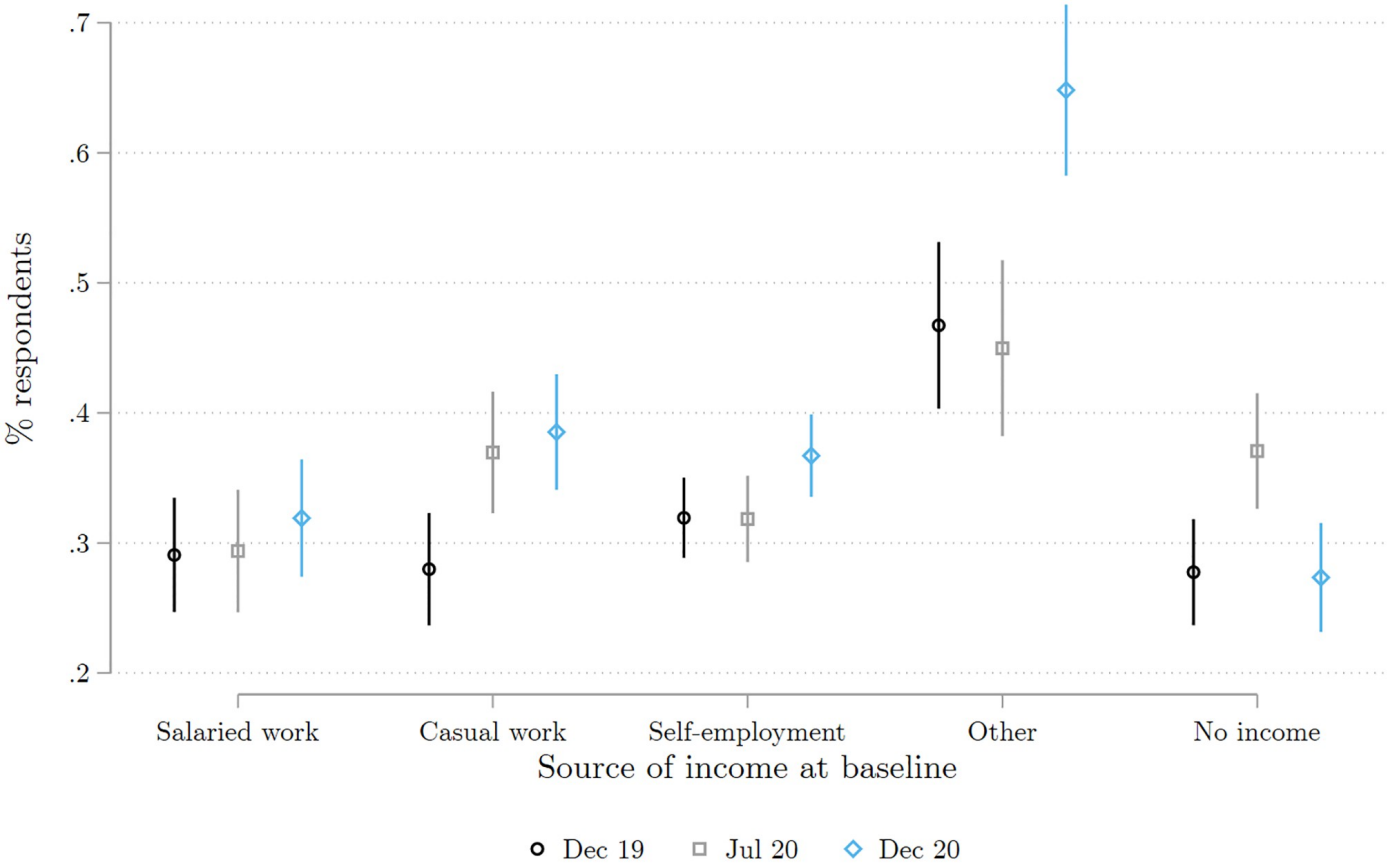
Recipients of private transfers, by main income source in December 2019. Each marker represents the proportion of respondents who report receiving at least one transfer from a private source over a specified recall period. Each proportion is calculated by regressing on survey dummies a dummy equal to one if the respondent reported receiving at least one transfer. The corresponding 95% confidence intervals are shown in color. The specified recall period for Dec 19 (the baseline survey) and Dec 20 (the second COVID survey) is the 12 months preceding the survey. For Jul 20 (the first COVID survey) the recall period is since the beginning of the pandemic in March 2020. The Figure combines all the respondents interviewed in either of the two COVID survey waves 1 and 2. Source: Baseline survey and COVID survey waves 1 and 2.

**Fig 13:**
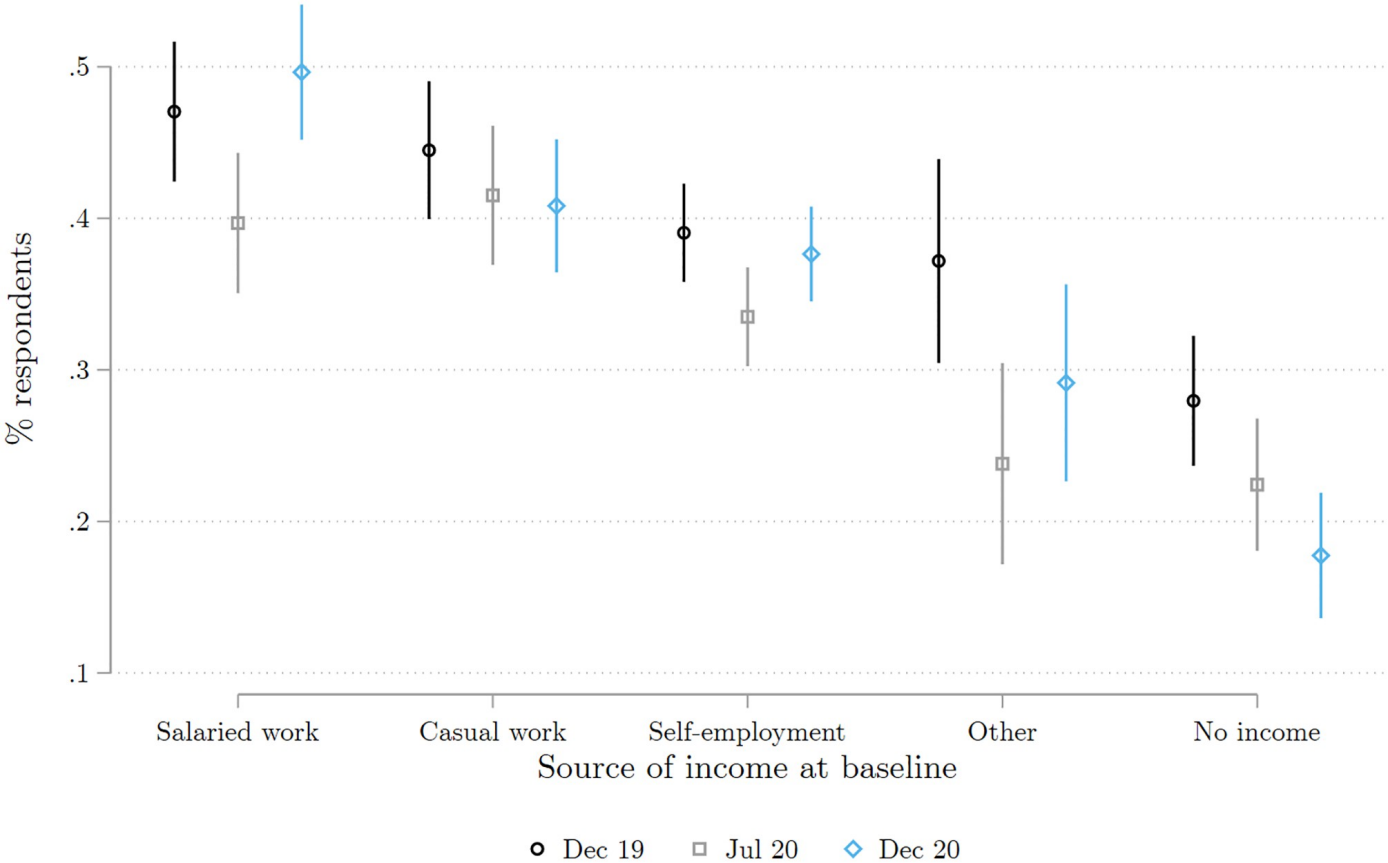
Respondents who sent or lent money, by main income source in December 2019. Each marker represents the proportion of respondents who report sending or lending money to other individuals over a specified recall period. Each proportion is calculated by regressing on survey dummies a dummy equal to one if the respondent reported making at least one transfer to another individual. The corresponding 95% confidence intervals are shown in color. The specified recall period for Dec 19 (the baseline survey) and Dec 20 (the second COVID survey) is the 12 months preceding the survey. For Jul 20 (the first COVID survey) the recall period is since the beginning of the pandemic in March 2020. The Figure combines all the respondents interviewed in either of the two COVID survey waves 1 and 2. Source: Baseline survey and COVID survey waves 1 and 2.

Regarding private transfers, we find in Fig 12 that the proportion of respondents who report receiving a transfer in the last 12 months is higher in December 2020 than December 2019). This increase occurred in spite of the fact that the likelihood of transfers from *abroad* (i.e., remittances) dropped in 2020, as shown in S4 Fig. Given that most transfers recorded at baseline take place between respondents and other individuals in the country, it is possible that Abidjan residents relied on assistance from friends and family outside of the metropolis. Unfortunately we do not have information on the origin of the domestic transfers received by our respondents. We also note that non-employed and casual workers are those most likely to report receiving private transfers, suggesting that these transfers were, on average, reaching those most in need. In parallel, Fig 13 indicates that the proportion of respondents who report sending or lending money to others dropped during 2020 and had not fully recovered by December 2020.

Turning to government transfers, we start by noting that they were virtually non-existent at baseline: less than 1% of baseline respondents declare receiving a transfer from the government. In the first COVID survey, we asked respondents about specific in-kind government transfers that were advertised in the media, such as face masks, antiseptic gel, money, and food. Survey responses indicate that only 5.4% of respondents recall receiving such goods. Table 2 breaks this down by income quartiles—keeping in mind that, by design, our sampling frame does not include the richer segments of the population. We see that 8% of respondents in Q4, the highest income quartile in our sample, report receiving face masks and antiseptic gel, compared to 2.5% of the lower quartiles Q1. Salaried and self-employed respondents—who tend to have a higher income on average—similarly report having received these products more often than others.

**Table 2:** Beneficiaries of government support programs.

	N	Overall Average	Income Quartiles June 2020				Employment June 2020			
			Q1	Q2	Q3	Q4	Salaried	Casual	Self-employed	Not employed
**Panel A: Monetary or in-kind transfer**										
Monetary or in-kind transfers at baseline	2343	0.4	0.4	0.4	0.5	0.0	0.3	0.3	0.2	0.7
Distribution of masks/gel Apr-Jul 2020	2343	5.4	2.5	5.1	6.9	8.0	7.8	3.5	7.0	4.3
Distribution money/food Apr-Jul 2020	2343	3.0	2.7	3.3	3.2	2.4	2.8	3.8	2.7	3.1
**Panel B: reduction of electricity bill**										
Full sample	2343	11.3	10.1	11.6	12.5	11.4	14.8	13.1	10.7	10.0
Sample with legal access to electricity	1680	14.3	13.4	14.9	16.3	13.5	18.4	16.8	13.5	13.0
Sample eligible for reduction	384	38.3	32.8	40.6	40.2	37.2	41.9	39.1	43.2	33.1

*Notes*: The Table presents the percentage of respondents who report having benefited from government support programs. Each row in Panel A corresponds to a different government support program. Each row in Panel B corresponds to a different sample. The full sample includes all the respondents interviewed in COVID survey wave 1. The sample with legal access to electricity is restricted to the 1680 respondents who have a contract with the government electricity supplier. The 384 respondents eligible for a reduction includes those who satisfy the conditions for benefiting from a reduction in their electricity bill. The first column includes all income and employment categories. Columns Q1 to Q4 break down the samples by quartiles of individual income reported in COVID survey wave 1 for June 2020 (N = 2,224). Columns ‘Salaried’ to ‘Not employed’ breaks down the sample by the type of main source of income reported in COVID survey wave 1 for June 2020 (N = 2,194). Source: Baseline survey and COVID survey wave 1.

Regarding government transfers of cash or food, respondents in the second quartile are more likely to report receiving government transfers (3.2%), compared to 2.7% of those in quartiles 1, and almost equally likely compared to 3.3% of those in the third quartile. Casual workers and not-employed benefited the most from this type of aid, but the proportion of respondents who received transfers remains low across all categories. From this we conclude that government transfers only benefited a small fraction of our relatively poor sample population, and that these transfers were not particularly targeted towards either the most vulnerable households or those most negatively affected by lockdown.

Other COVID policies were introduced by the authorities. In particular, the government announced that it would subsidize electricity, a measure similar to that taken in Ghana [33]. The subsidy was targeted towards poorer households: it was limited to those with an electricity supply limited to 5 Amp (e.g., insufficient to power a large fridge) and with a prepaid plan from the electricity supplier, since these consumers are typically poorer than those with a post-paid plan. Based on these criteria, 23.3% of the households in our study sample can be considered as eligible. Survey responses, however, indicate that only a third of these eligible respondents received a reduction in their electricity bill from the government. Respondents in the upper echelons of income in our sample seem to have benefited as much than poorer respondents—if not more. Whatever the reason, what our results show is that, in spite of being designed to favor the poor, this intervention often failed to reach its intended target.

### 4.6 Child schooling

Schools in Côte d’Ivoire were closed from April to August 2020, but they reopened in September 2020. One of the preoccupations of policy makers regarding the potential long-term impact of the pandemic is the risk that prolonged school closure would lead to school drop-out, especially among adolescent girls. To shed light on this, this Section documents schooling outcomes for the children of our respondents.

In the baseline survey, we asked questions about schooling for all children between 6 and 16 years of age. Information was collected from 1,147 households on 2,094 children with an average (and median) age of 10. Table 3 shows the net enrollment rate for these children just before the pandemic (start of academic year 2019–2020). It is very high (93%), a figure consistent with the World Bank estimate of around 90% at the national level in Côte d’Ivoire.

**Table 3:** School enrollment.

Sample	Overall	Gender		Age category		
		Boys	Girls	[6-9]	[10-13]	[14-16]
Enrolled in 2019-2020	93.0	92.7	93.4	93.3	94.1	90.9
Enrolled in 2020-2021	91.6	91.3	92.0	94.1	92.3	85.9
Not enrolled in Sept 2020 because:						
School was closed	0.7	0.9	0.4	0.6	0.6	0.9
Child not willing to go to school	2.7	2.3	3.3	1.8	3.4	3.5
Child finished school	0.5	0.7	0.3	0.4	0.4	0.9
Child needs to work/help at home	2.2	2.6	1.7	0.8	1.9	5.3
No money to pay for school	1.2	1.3	1.2	1.3	0.7	1.8
**Observations**	2,094	1,087	1,007	864	761	469

*Notes*: The first two rows of the Table compare average enrollment rates in academic years 2019–2020 and 2020–2021. Enrollment is a binary variable equal to 1 if the child is reported by parents as going to school, irrespective of grade. For 2020–2021, enrollment information is as reported in January 2021. The remaining rows of the Table reports the proportion of non-enrolled children, broken down by the main reason given by the respondent for the child not attending school. The first column includes the full sample of children. Columns 2 and 3 break down the sample by gender. The last three columns break down the sample by age of the child in January 2021. The sample includes 2,094 children aged 6–16 in January 2021, living in 1,113 surveyed households. Source: COVID survey wave 2.

In wave 2, we collected information on the enrollment rate of these same children in the 2020–21 academic year. In spite of the temporary school closure, enrollment rates upon reopening were very high (92%), with no differential dropout among girls. It is only for the oldest cohort, aged from 14 to 16 years, that enrollment rates seem to have suffered somewhat from the school closure, with a 5 percentage point drop from 91% pre-pandemic to 86% in January 2021. This decrease is mainly driven by children aged 16, old enough to be stopping school. For the few children out of school, we asked parents the reason why the child was no longer attending school. The main reasons given are either that the child no longer wants to go school or that the child needs to help or work at home.

Given the small decrease in enrolment rate and the fact that the lack of money to pay for school fees is only mentioned for 1% of the children, it appears that the loss of employment and fall in income have not negatively impacted the schooling enrollment of children in our sampled households. On the whole, these results suggest that enrollment did not suffer too much from the crisis, which is reassuring. But, given the lack of access to remote learning opportunities, it likely that children’s learning suffered substantially from the extended closure of schools, something we cannot document with the data at hand.

### 4.7 Health and well-being

In wave 1, we investigated the direct effect of COVID on the health of the respondents and their family. 28.6% of respondents report being ill in the three months preceding the survey and another 14.4% report a household member falling ill. These proportions are broadly comparable to those reported at baseline, when 72.3% of respondent reported at least one bout of illness over the preceding 12 months. Of those who were ill in the preceding three months, 12.8% respondents (86 individuals) and 10.2% household members (49 individuals) took a COVID test. Among those, only 3 individuals (2.2%) tested positive—confirming that COVID-19 was probably not highly prevalent in Abidjan at the time.

The impact of COVID-19 and the government response to it could nonetheless have affected the psychological well-being of the population. The fear of infection, the large negative income shocks incurred by many, or social distancing measures could all lead to an increase in stress, anxiety, and loneliness. To investigate this possibility, we collected information on well-being during our baseline and wave 2 surveys.

**Fig 14:**
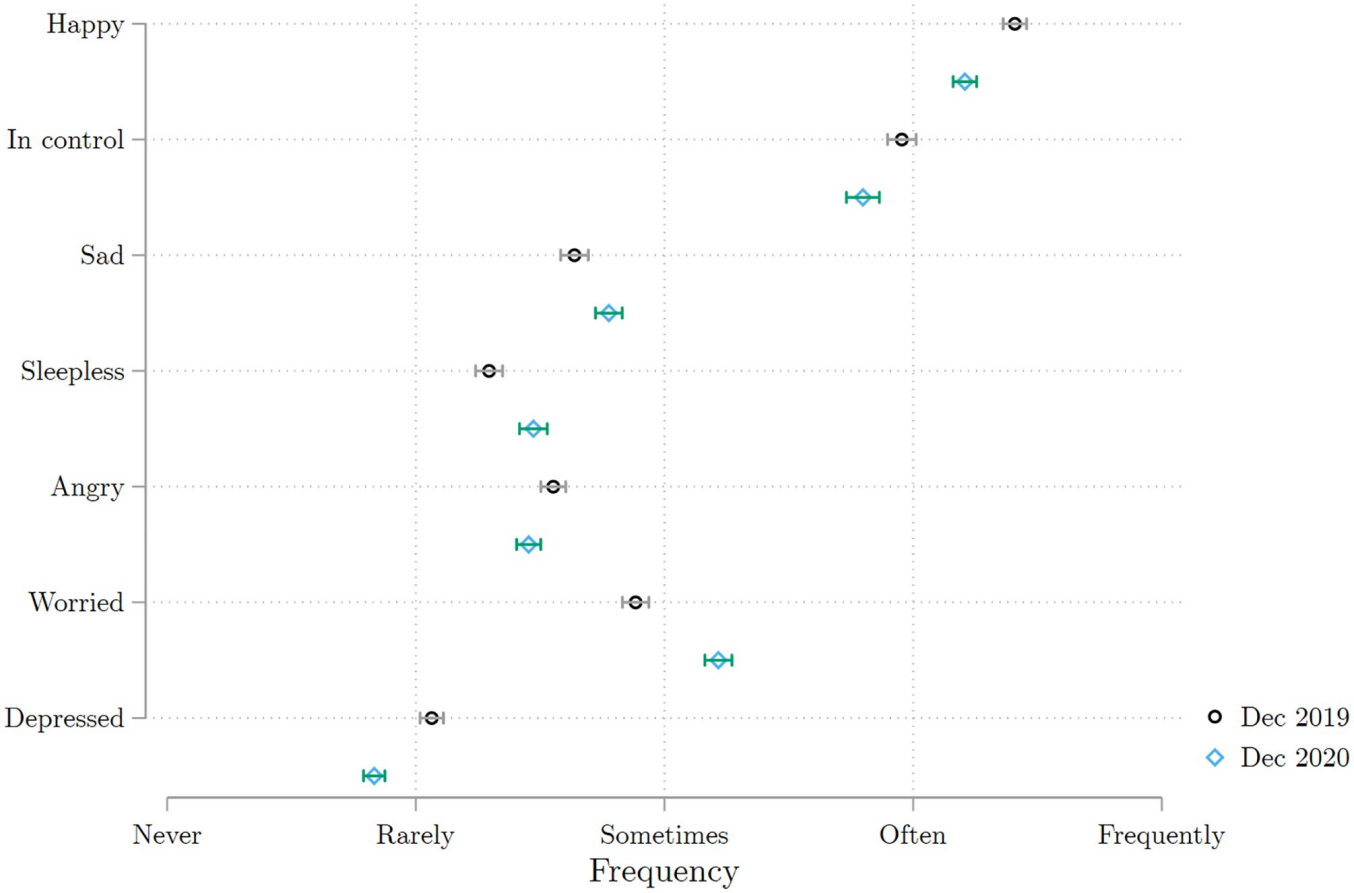
Subjective well-being indicators, by survey wave. Each line presents the average value of the response to a separate Likert scale question on one aspect of emotional well-being, summarized by the label on the left. The scale ranges from 1 (Never) to 6 (Always). The questions were asked in the baseline survey and in COVID survey wave 2. The Figure includes all the individuals interviewed in COVID wave 2. Source: Baseline survey and COVID survey wave 2.

Fig 14 presents the frequency of various emotions as experienced and reported by respondents. We find that respondents are more likely to report feeling sad or worried after than before the pandemic. They also report having more trouble sleeping and being less in control, and they report positive feelings less frequently. We also note that the small fraction of respondents who reported being depressed in December 2019 got even smaller in December 2020. We do not know why this is the case: perhaps people described themselves now more as ‘worried’ than ‘depressed’. Given that depression is often stigmatized in much of Africa, another possibility is that those few individuals who admitted being depressed in 2019 found themselves better able to receive psychological support in the middle of the pandemic, when everyone was facing difficulties. These findings echo those of other studies in other settings that have documented the fall in mental health following population containment policies in LMICs—e.g.: [9, 24–27] for adults; and [34] for children.

## 5 Discussion and conclusions

This paper has documented how the COVID pandemic disrupted the income earning activities of poor household’s living in the city of Abidjan and its surroundings. Our data structure provides panel information from December 2019 to December 2020, covering the immediate pre-COVID period and the first year of the pandemic. The wide range of question asked during the baseline allowed us to explore many dimensions of heterogeneity. We do not, however, have prior information on possible seasonal patterns in employment, income, and consumption for the months most affected by the pandemic. This important caveat needs to be kept in mind when interpreting our findings.

Given the available estimates of the number of COVID infections and COVID-related deaths in the first year of the pandemic, the pandemic appears to have had very limited impact on health in Côte d’Ivoire and other African countries other than South Africa. This did not stop many African government from introducing strict lockdown measures in the early stages of the pandemic, at a time where they number of local cases was extremely small. As a result of these temporary measures and subsequent fear of the illness, personal mobility was temporarily reduced in Côte d’Ivoire as it was in many parts of Africa—although by not as much as other parts of the world.

In spite of this, our findings document large changes in employment that are consistent with our sample population’s limited access to stable jobs. We observe a large decrease in employment among salaried, casual, and self-employed workers between March and June 2020 and we note a drastic fall in income during the same period. The reduction in income seems to affect our sampled population at large—e.g., irrespective of educational level or commuting time at baseline. By December 2020, however, much recovery had taken place, with most respondents regaining a form of employment similar to what they had at baseline. A similar picture emerges regarding the effect of lockdown and other restrictions on food consumption: we observe a commensurate fall in consumption up to July 2020 but, by December 2020, respondents had, on average, come back to their pre-COVID consumption level.

One channel through which respondents sought to weather the crisis was private transfers coming from other parts of the country, at a time when remittances from abroad also fell. Government policies aimed at alleviating the worst effects of lockdown seem to have only reached a few people, and not necessarily those most in need. Whether interventions could have been better targeted is unclear. A study, [35], indeed shows that identifying the poor in Greater Abidjan is far from an easy task without undertaking an in-depth survey which, by definition, would not have been feasible given the available time frame and the travel restrictions themselves.

Child schooling seems to have suffered little from the school closures that were introduced in Côte d’Ivoire at the beginning of the pandemic: enrollment rates in December 2020 had bounced back to the high level they had at baseline, with limited evidence of drop-outs. We cannot rule out the possibility of a large learning loss, but we have no data to speak to that issue.

While we observe that respondents had, on average, recovered by December 2020 in terms of employment, income, expenditure, and school enrollment, it remains that the lockdown and other restrictions introduced at the beginning of the pandemic had a very large negative effect on several dimensions of economic welfare. It is not for us to tell whether this welfare cost was justified in terms of health policy—we cannot be sure of how the pandemic would have evolved in Côte d’Ivoire without the restrictions imposed by the government. What is clear, however, is that the cost was large and significant and that it affected a large fraction of society, including many of the poor. It also appears that, while many of our respondents received financial assistance through private transfers from individuals in other parts of the country, the targeted policies that the government introduced to limit the economic incidence of the crisis did not have much of an impact and were, by and large, not targeted towards those most in need.

## Supporting information

S1 Fig Changes in the proportion of respondents who report earned income. Notes: Each map is divided into hexagons for the purpose of this analysis. Hexagons that appear in grey are not in the sample. Colored hexagons are those containing sampled respondents, whose responses are averaged within each hexagon to create the maps. The names of the relevant municipalities have been overlaid on the maps to facilitate interpretation. Respondents located in outlying areas are omitted from the maps and only the center of Fig 1 is shown for readability. White areas indicate either the presence of a water body (e.g., ocean, lagoon) or non-coverage by the study. See Fig 1 for more details about the geography. Source: Baseline and COVID survey waves 1 and 2.
(TIF)

S2 Fig Average weekly food expenditure, by location of residence at baseline. Each whisker plot presents the average of household weekly expenditures reported in each of the three survey waves, broken down by the respondent’s location of residence at baseline. Each average is calculated by regressing reported expenditures on wave dummies and is shown by the marker. The corresponding 95% confidence intervals are shown as vertical lines. The Figure combines the respondents interviewed in COVID survey waves 1 and 2. To correct for outliers, the top 1% of the weekly expenditure is winsorized. Winsorized means that values above the top percentile are replaced by the value of the 1% percentile. Source: Baseline survey and COVID survey waves 1 and 2.
(TIF)

S3 Fig Kernel density of food expenditures, by location of residence at baseline. The Figure shows the estimated Kernel density plot of the household weekly food expenditures reported in each of the three survey waves. Each panel includes the respondents in that residential location at baseline. The Figure combines the respondents interviewed in COVID survey waves 1 and 2. To correct for outliers, the top 1% of the weekly expenditure is winsorized. Source: Baseline survey and COVID survey waves 1 and 2.
(TIF)

S4 Fig Proportion of respondents who received transfers from abroad, by employment status in December 2019. Each whisker plot represents the proportion of respondents who report receiving transfers from abroad. This information was only collected at baseline and wave 2 and, in both cases, it covers the 12 months preceding the survey. Each marker shows the proportion calculated by regressing, on wave dummies, a dummy equal to one if the respondent reported receiving at least one transfer from abroad in the last 12 months. Only respondents interviewed in COVID survey wave 2 are included in the regression. The corresponding 95% confidence intervals are shown as vertical lines. Source: Baseline survey and COVID survey wave 2.
(TIF)

S1 File Sampling frame and listing. (PDF)

S2 File Non-response robustness checks. (PDF)
