# Supplementary figures and images for: Panel data evidence on the effects of the COVID-19 pandemic on livelihoods in urban Côte d’Ivoire
Source: PLoS One. 2023 Feb 1;18(2):e0277559. doi: 10.1371/journal.pone.0277559 (PMC9891504; doi:10.1371/journal.pone.0277559)

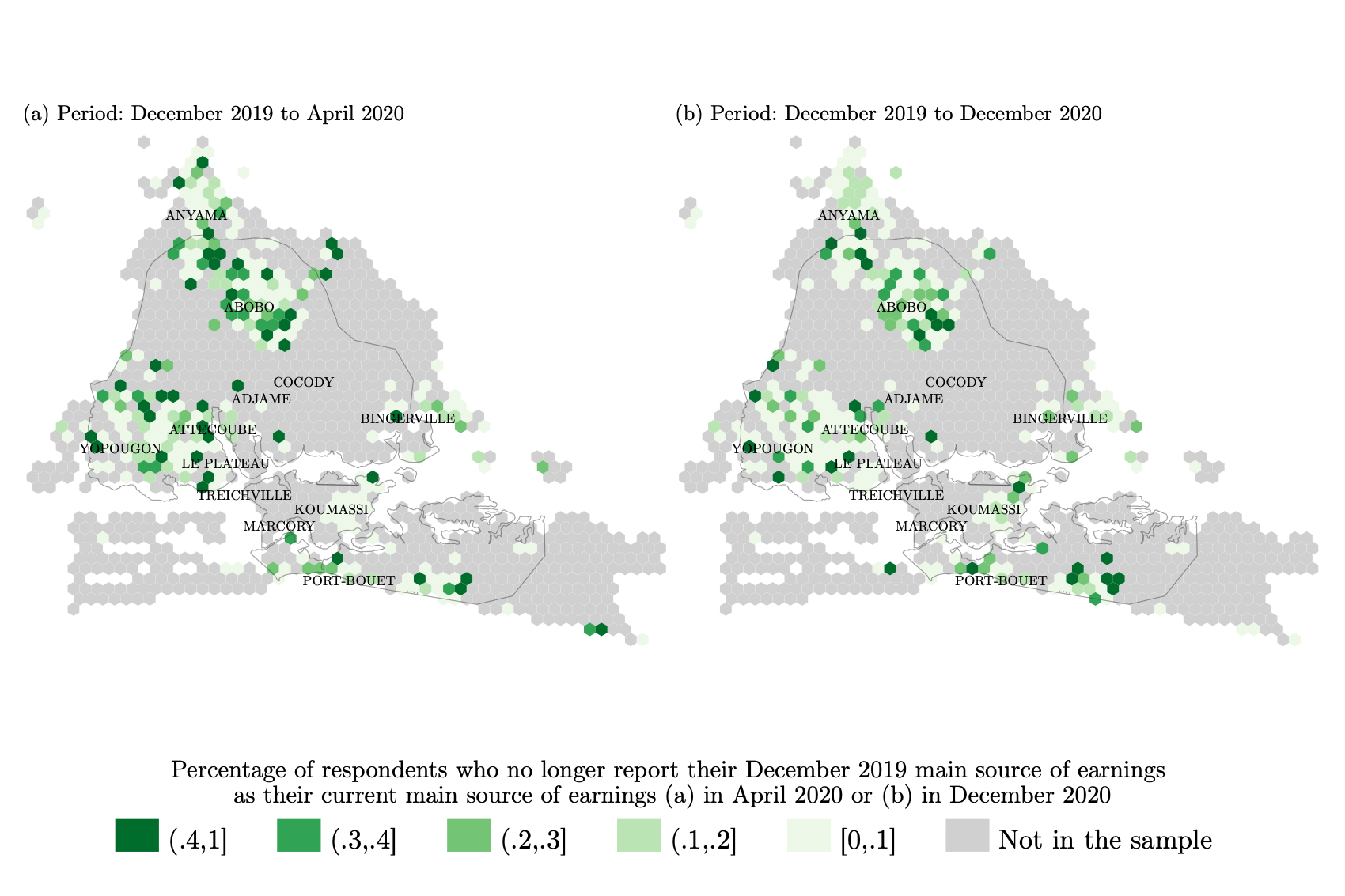

Supplement: S1 Fig — Notes: Each map is divided into hexagons for the purpose of this analysis. Hexagons that appear in grey are not in the sample. Colored hexagons are those containing sampled respondents, whose responses are averaged within each hexagon to create the maps. The names of the relevant municipalities have been overlaid on the maps to facilitate interpretation. Respondents located in outlying areas are omitted from the maps and only the center of Fig 1 is shown for readability. White areas indicate either the presence of a water body (e.g., ocean, lagoon) or non-coverage by the study. See Fig 1 for more details about the geography. Source: Baseline and COVID survey waves 1 and 2. (TIF) [file pone.0277559.s001.tif]

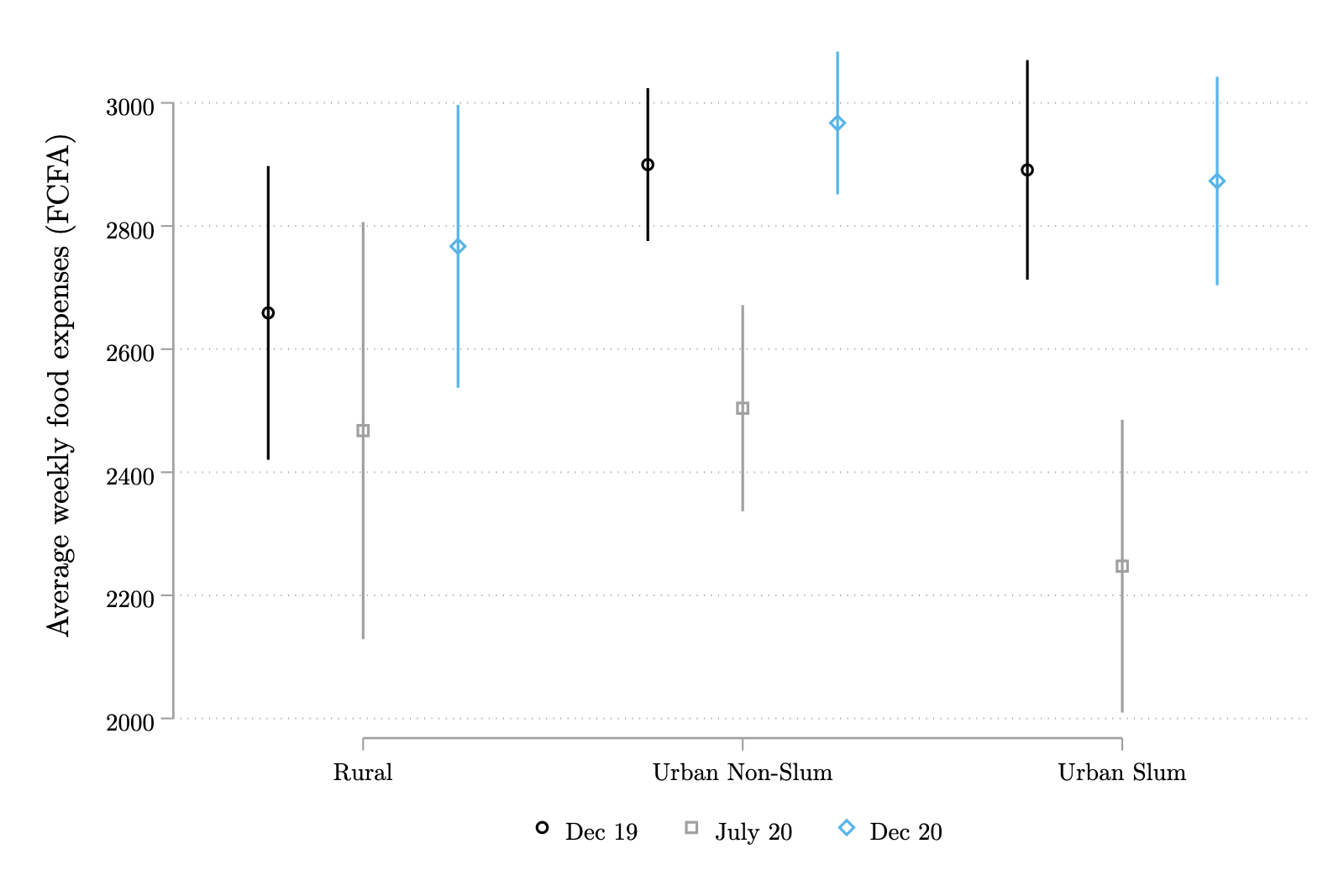

Supplement: S2 Fig — Each whisker plot presents the average of household weekly expenditures reported in each of the three survey waves, broken down by the respondent’s location of residence at baseline. Each average is calculated by regressing reported expenditures on wave dummies and is shown by the marker. The corresponding 95% confidence intervals are shown as vertical lines. The Figure combines the respondents interviewed in COVID survey waves 1 and 2. To correct for outliers, the top 1% of the weekly expenditure is winsorized. Winsorized means that values above the top percentile are replaced by the value of the 1% percentile. Source: Baseline survey and COVID survey waves 1 and 2. (TIF) [file pone.0277559.s002.tif]

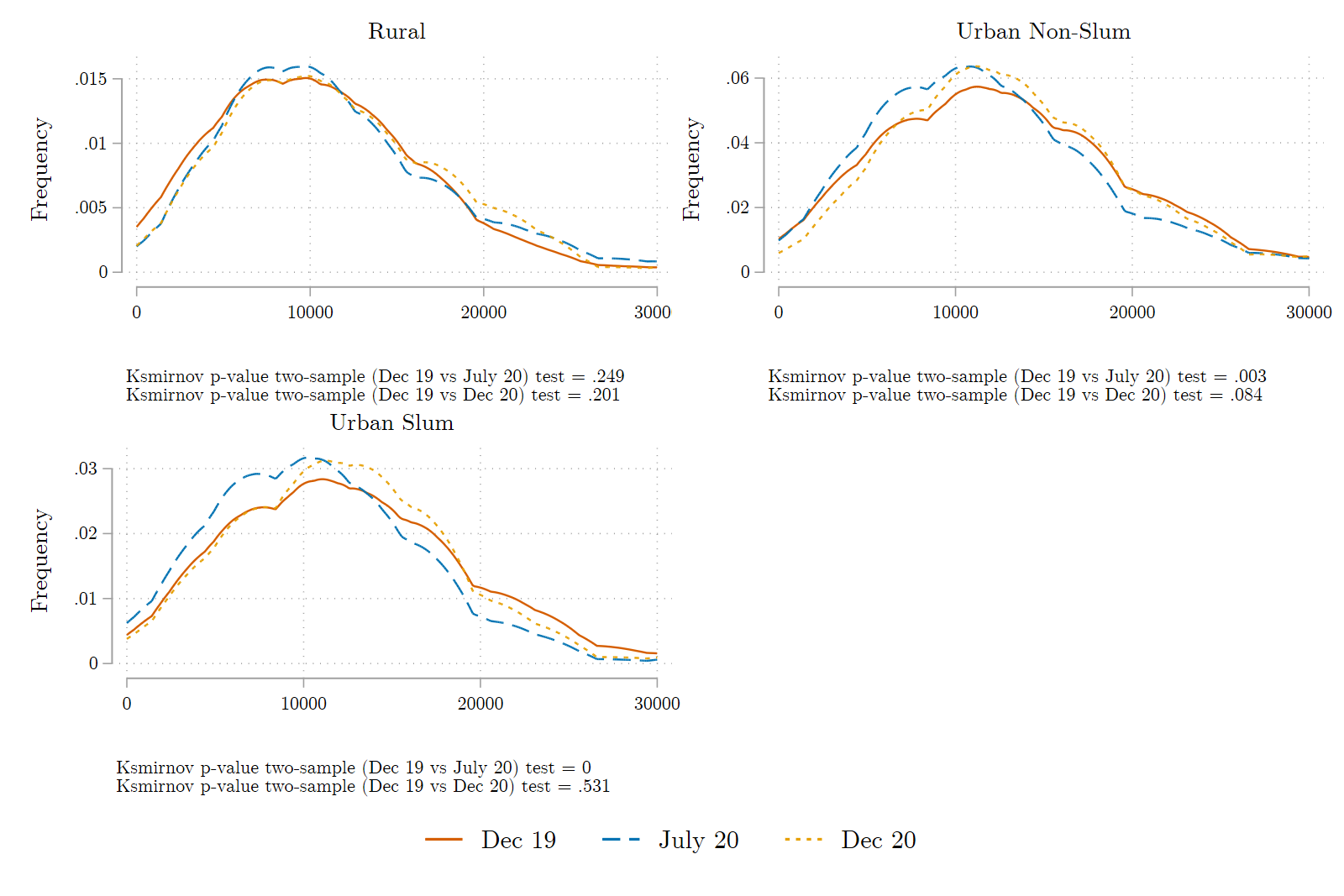

Supplement: S3 Fig — The Figure shows the estimated Kernel density plot of the household weekly food expenditures reported in each of the three survey waves. Each panel includes the respondents in that residential location at baseline. The Figure combines the respondents interviewed in COVID survey waves 1 and 2. To correct for outliers, the top 1% of the weekly expenditure is winsorized. Source: Baseline survey and COVID survey waves 1 and 2. (TIF) [file pone.0277559.s003.tif]

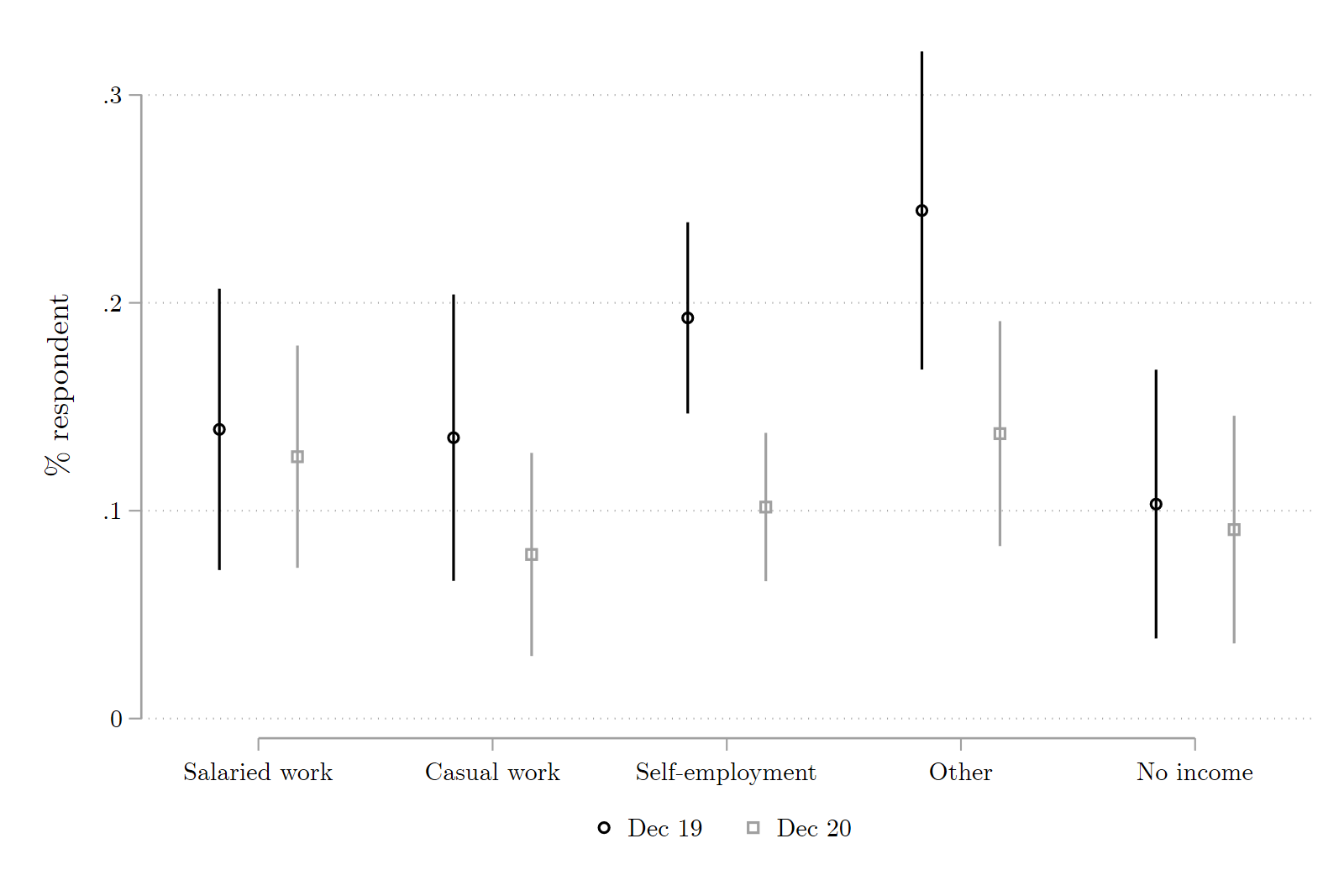

Supplement: S4 Fig — Each whisker plot represents the proportion of respondents who report receiving transfers from abroad. This information was only collected at baseline and wave 2 and, in both cases, it covers the 12 months preceding the survey. Each marker shows the proportion calculated by regressing, on wave dummies, a dummy equal to one if the respondent reported receiving at least one transfer from abroad in the last 12 months. Only respondents interviewed in COVID survey wave 2 are included in the regression. The corresponding 95% confidence intervals are shown as vertical lines. Source: Baseline survey and COVID survey wave 2. (TIF) [file pone.0277559.s004.tif]
