# Supplementary material for: Panel data evidence on the effects of the COVID-19 pandemic on livelihoods in urban Côte d’Ivoire
Source: PLoS One. 2023 Feb 1;18(2):e0277559. doi: 10.1371/journal.pone.0277559 (PMC9891504; doi:10.1371/journal.pone.0277559)
Supplement: S1 File — (PDF) [file pone.0277559.s005.pdf]

S5 File: Sampling

Area selection

The main objective of the AUDRI project is to create a representative sample of the peri-urban poor population of the Abidjan district, plus some nearby areas. The 2015 Japan International Cooperation Agency (JICA) map was used to identify which municipalities or sous-prefectures to target. The district of Abidjan contains ten stand-alone municipalities (Cocody, Adjamé, Treichville, Plateau, Marcory, Abobo, Attecoube, Koumassi, Port-Bouet, and Yopougon – collectively refereed to as Abidjan City) plus four sous-prefectures (Anyama, Bingerville, Brofodoume, and Songon) that are partly rural (see Fig 1). As per Ivorian nomenclature, a municipality is 100% urban while a sous-prefecture (i.e., sub-district) includes both an urban municipality with its administrative center, and a collection of villages classified as rural. Most of our sample comes from the Abidjan district, but we omit the five municipalities that occupy the center of the city (Cocody, Adjamé, Treichville, Plateau, and Marcory) and we focus instead on the outlying municipalities where poorer households tend to reside. To these areas we add the sous-prefectures of Bonoua and Grand Bassam in the Comoe District, and the sous-prefectures of Azaguie, Alepe, Oghlwapo, Dabou and Jaqueville from the District of Lagunes. In these sous-prefectures we focus primarily on the more urbanized areas, but we also include a number of villages which constitute our 'rural' sample. Together, all these administrative units constitute what we refer to Greater Abidjan for the purpose of this study.

Within these selected administrative units, we use the Institut National de Statistique (INS)'s enumerations areas (EAs) as building blocks for our sample frame. In 2014, EAs were defined as follows: (i) in urban area, a EA includes exactly 200 households; and (ii) in rural areas, an EA includes all households living in a village. For each selected municipality, we used the definition of EAs to estimate their total population. Based on these estimates, 85% of the population of Greater Abidjan (as defined for this project) lives in Abidjan City and 93% live in urban municipalities. Because AUDRI's geographical focus is on areas where urbanisation is most likely to increase in the coming years, we designed the AUDRI sampling frame to have 50% of the listed households in Abidjan City and the rest outside. This resulted in having 78% of the listed households living in urban areas, in and around Abidjan City. This methodology resulted in a sampling frame consisting of 84 selected villages randomly selected from 11 sous-prefectures, plus 622 urban EAs randomly selected from the same 11 sous-prefectures and the 5 municipalities selected in Abidjan City. No stratification was used for urban EAs since, by construction, they all have approximately the same population and equal sampling results in a balanced unweighted sample. For rural EAs, since the number of households in a village varies, we use a sampling method that endeavors to be proportional to the population of the village.

Listing

The listing exercise was launched in mid-July 2019 and lasted almost 2 months. During the listing, we collected information about each member of the household, assets ownership, health and CMU enrollment. Respondents who were aged 18 and above and gave their consent to be interviewed were surveyed. To randomize which households were listed, enumerators followed the well-established method of starting from the centroid[1] of the urban EA and knocking on every 20 doors, counting from the closest door to the centroid. In rural EAs where dwellings are scattered over the landscape, enumerators targeted a random number of doors until the target sample size was reached, or until no other dwelling was found.

The initial objective was to list 8000 households in and around Abidjan within the 84 villages/rural EAs and 622 enumeration areas (EA) that were identified as sampling frame. From this initial base, we dropped 16 villages that were too difficult to reach, 23 villages because of administrative constraints (e.g., the local chief or administrator refused for the village to participate to the survey), 9 urban EAs because they turned out to be empty (e.g., slum expulsions), 4 urban EA that was too insecure, and 1 urban EA that was inaccessible. This led to a reduction of our estimated sample size by 954 households. We also found that, in numerous villages, the number of inhabitants was smaller than anticipated. This resulted in an additional reduction of listing sample by 752 households. Overall, 6294 households were identified and targeted by the listing exercise. Among these 6294 households, we suffered a refusal rate of 7% and an absence rate of 11% (we were expecting 1 in 10 absent

[1]The centroid was computed using GIS and the shapefiles provided by INS.

households, based on estimates for the region). The listing's response rate was 82%, resulting in a total listing sample of 5161 households located in 608 urban EAs and 45 villages/rural EAs.

Baseline sample

The sample for the wave 0 survey was constructed by first randomly selecting 70% of these listed households in each EA, and then randomly selecting one adult per selected household. To avoid oversampling individuals from singleton households, we pooled all singleton households (N=11) together and sampled 70% of them. Because EAs are constructed to have roughly the same population, the sample is self-weighting, which means we did not use sampling weights when randomly selecting participants among listed households. In rural EAs, the number of listed households is roughly proportional to the number of households residing there. This resulted in a total baseline sample of 2940 individuals.
