# Supplementary material for: Panel data evidence on the effects of the COVID-19 pandemic on livelihoods in urban Côte d’Ivoire
Source: PLoS One. 2023 Feb 1;18(2):e0277559. doi: 10.1371/journal.pone.0277559 (PMC9891504; doi:10.1371/journal.pone.0277559)
Supplement: S2 File — (PDF) [file pone.0277559.s006.pdf]

S6 File: Robustness to Attrition

Figure A.1: Fraction of respondents who do not report income from working as their main source of income

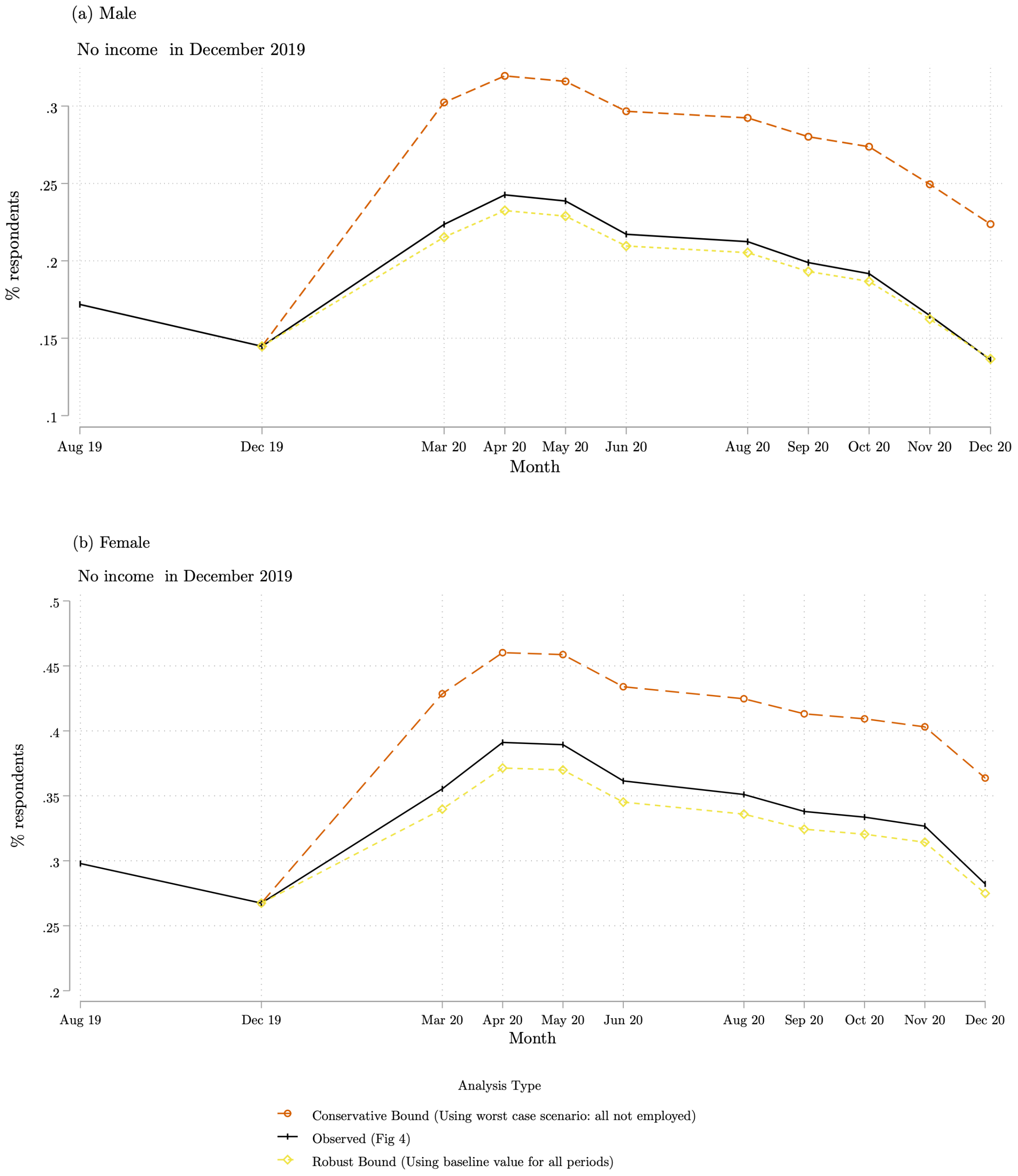

Notes: Each graph presents the point estimates of month dummies for three separate regression. One regression is based on actual data, using respondents to Covid survey waves 1 and 2 ('Observed'). The other regressions add observations for the individuals who were identified as part of the targeted sample 2161 individuals but did not take part in the wave 1 and 2 surveys. In the 'conservative' scenario, these added observations all assume that non-respondents are no longer employed, and therefore no longer report any earned income. In the 'robust' scenario, these added observations assume that non-respondents keep the employment status they had in the wave 0 survey. The dependent variable is 1 if the (actual or assumed) respondent reports no earned income in each month, and 0 otherwise.

Figure A.2: Number of days worked per week, by main source of income at baseline

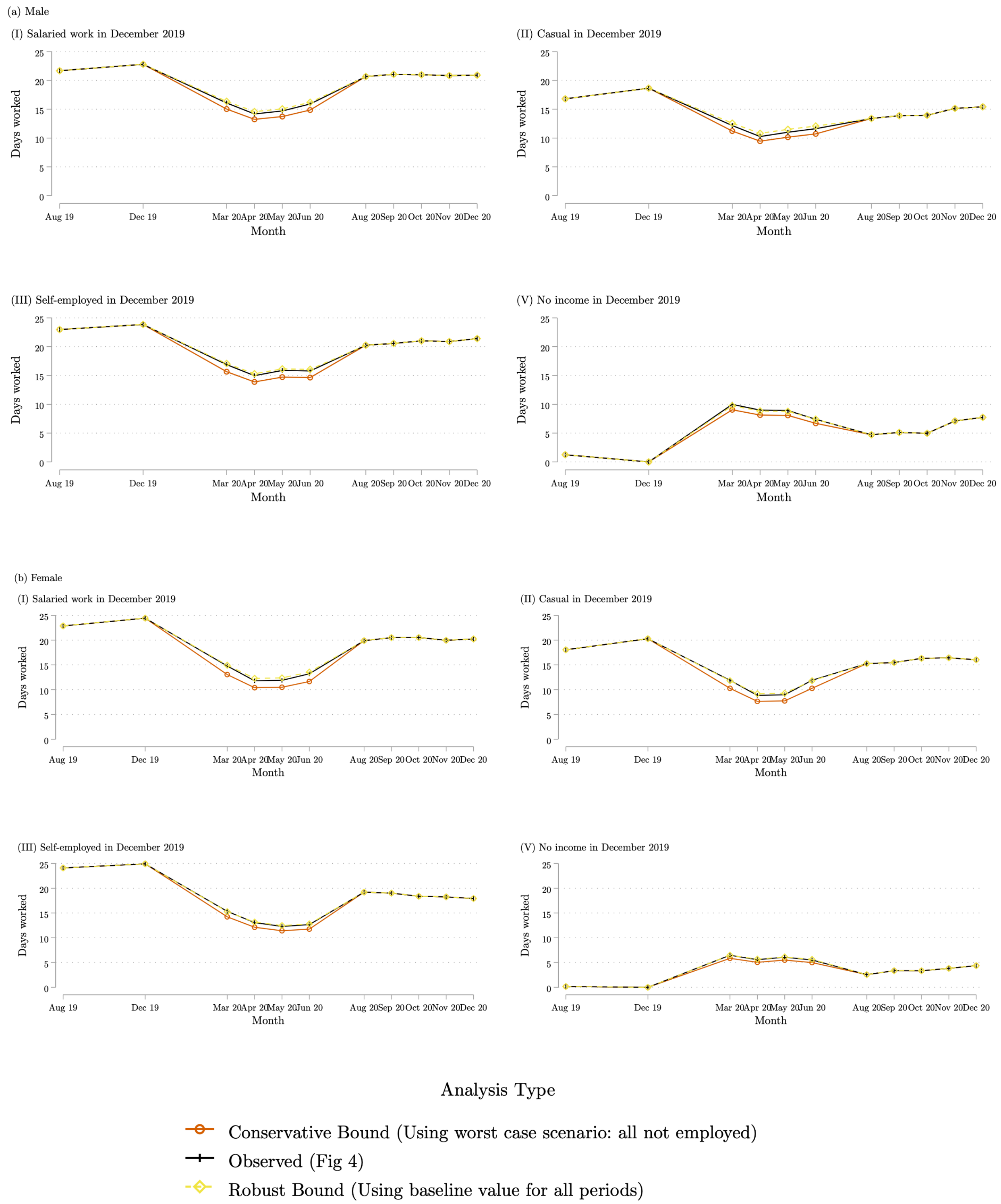

Notes: Each graph presents the point estimates of month dummies for three separate regression. One regression is based on actual data, using respondents to Covid survey waves 1 and 2 ('Observed'). The other regressions add observations for the individuals who were identified as part of the targeted sample 2161 individuals but did not take part in the wave 1 and 2 surveys. In the 'conservative' scenario, these added observations all assume that non-respondents are no longer employed, and therefore work zero hours per week. In the 'robust' scenario, these added observations assume that non-respondents keep the employment status they had in the wave 0 survey – and thus the same number of worked days per week as in wave 0. The dependent variable is the number of days worked per week in each month. For simplicity, we omit the graph relative to individuals who reported non-zero unearned income as their main source of income in December 2019.

Figure A.3: Individual monthly income, by main source of income at baseline

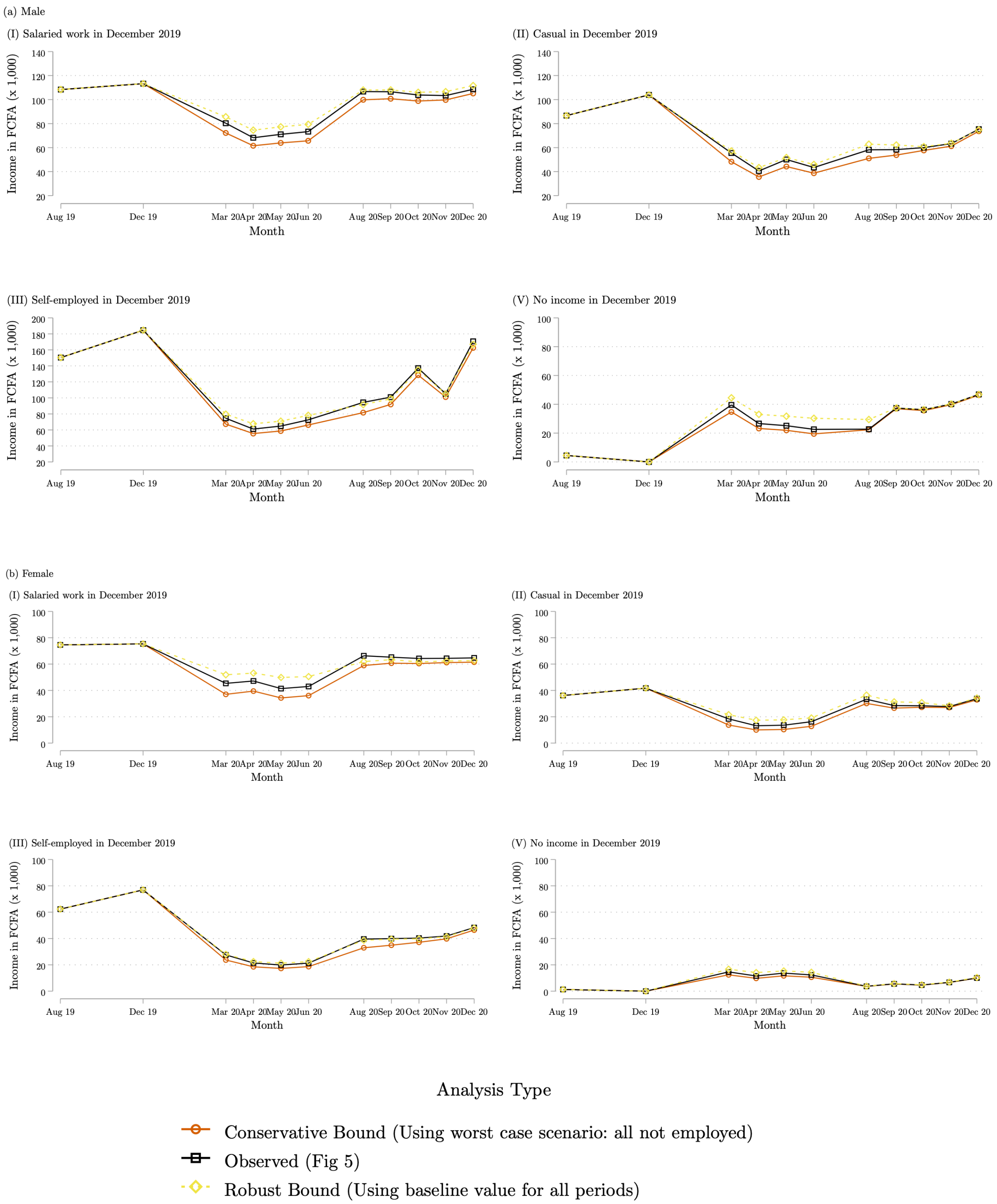

Notes: Each graph presents the point estimates of month dummies for three separate regression. One regression is based on actual data, using respondents to Covid survey waves 1 and 2 ('Observed'). The other regressions add observations for the individuals who were identified as part of the targeted sample 2161 individuals but did not take part in the wave 1 and 2 surveys. In the 'conservative' scenario, these added observations all assume that non-respondents are no longer employed, and therefore no longer report any earned income. In the 'robust' scenario, these added observations assume that non-respondents keep the employment status they had in the wave 0 survey – and thus the same income. The dependent variable is the monthly income reported by the surveyed individual. For simplicity, we omit the graph relative to individuals who reported non-zero unearned income as their main source of income in December 2019.
